# Muscle and cardiac therapeutic strategies for Duchenne muscular dystrophy: past, present, and future

**DOI:** 10.1007/s43440-020-00134-x

**Published:** 2020-07-20

**Authors:** Agnieszka Łoboda, Józef Dulak

**Affiliations:** Department of Medical Biotechnology, Faculty of Biochemistry, Biophysics and Biotechnology, Jagiellonian University, Kraków, Poland

**Keywords:** Duchenne muscular dystrophy, DMD, Gene therapy, Cell therapy, Induced pluripotent stem cells, CRISPR/Cas9

## Abstract

**Background:**

Duchenne muscular dystrophy (DMD) is a severe X-linked neuromuscular childhood disorder that causes progressive muscle weakness and degeneration and results in functional decline, loss of ambulation and early death of young men due to cardiac or respiratory failure. Although the major cause of the disease has been known for many years—namely mutation in the *DMD* gene encoding dystrophin, one of the largest human genes—DMD is still incurable, and its treatment is challenging.

**Methods:**

A comprehensive and systematic review of literature on the gene, cell, and pharmacological experimental therapies aimed at restoring functional dystrophin or to counteract the associated processes contributing to disease progression like inflammation, fibrosis, calcium signaling or angiogenesis was carried out.

**Results:**

Although some therapies lead to satisfying effects in skeletal muscle, they are highly ineffective in the heart; therefore, targeting defective cardiac and respiratory systems is vital in DMD patients. Unfortunately, most of the pharmacological compounds treat only the symptoms of the disease. Some drugs addressing the underlying cause, like eteplirsen, golodirsen, and ataluren, have recently been conditionally approved; however, they can correct only specific mutations in the *DMD* gene and are therefore suitable for small sub-populations of affected individuals.

**Conclusion:**

In this review, we summarize the possible therapeutic options and describe the current status of various, still imperfect, strategies used for attenuating the disease progression.

## Duchenne muscular dystrophy: an overview

**Fig. 1:**
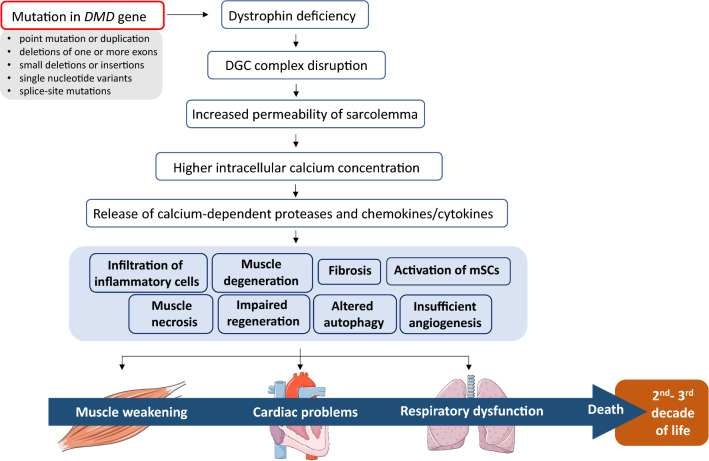
Complications in DMD. Various mutations in the *DMD* gene lead to dystrophin deficiency. A lack of functional dystrophin causes sarcolemmal disruption and calcium channel activation by mechanical stress. In turn, increased intracellular calcium level activates the release of calcium-dependent proteases and chemokines/cytokines, causing muscle degeneration, and necrosis. Other processes, including the activation of satellite cells (muscle stem cells, mSCs), impaired regeneration, increased inflammation, altered autophagy, and insufficient angiogenesis as well as augmented fibrosis are the hallmark of the disease. Progressive muscle weakening, together with respiratory and cardiac complications, leads to patients’ death in the 2nd to 3rd decades of their life. *DGC* dystrophin–glycoprotein complex

Duchenne muscular dystrophy (DMD, OMIM#310200) is a progressive, incurable, X-linked genetic disease that affects 1 in 5000–6000 boys. The disease is caused by the lack of functional dystrophin, due to over 7000 patient-specific mutations in *DMD*, one of the largest human genes containing 79 exons and approximately 2.4 million bp [1, 2]. Interestingly, in healthy skeletal muscle, dystrophin accounts for only 0.002% of total muscle protein, but its absence leads to tremendous detrimental effects on muscle functionality [3]. The protein links the actin cytoskeleton to the extracellular matrix in muscle fibers by forming interactions with subsarcolemmal actin and the large oligomeric dystrophin–glycoprotein complex (DGC) and regulates the proper functioning of muscle fibers (Fig. 1). The absence of dystrophin weakens the link between the sarcolemma and the actin cytoskeleton, resulting in membrane instability and muscle cell death. DGC is required, among others, for maintaining calcium (Ca^2+^) homeostasis [4] and for proper neuronal nitric oxide synthase (nNOS) activity and NO signaling muscle cells [5–7]. When DGC assembly is impaired, the increase in intracellular Ca^2+^ ion concentration activates Ca^2+^-dependent proteases, such as calpain and various chemokines and cytokines. This results in the continuous cycles of muscle degeneration and regeneration, the activation of satellite cells (muscle stem cells, mSCs), accumulation of inflammation, fibrosis, and increased oxidative stress, and leads to progressive muscle weakening and loss of muscle mass and function. Mislocalization of nNOS at the sarcolemma and disturbed NO homeostasis leads to impaired muscle blood flow and severe muscle fatigue. Additionally, DGC, by providing a scaffold for different proteins, plays a crucial role in numerous signaling pathways [1]. Of note, recent studies have also indicated that perturbations in other processes, such as mitochondrial signaling, autophagy, and angiogenesis, contribute to DMD progression [8]. These complications may be visible not only in the skeletal and cardiac muscles of DMD patients but also in the frequently used murine (e.g., *mdx* mice [9–11]) and canine (e.g., golden retriever muscular dystrophy; GRMD [12, 13]) models of DMD.

DMD patients can be diagnosed upon a thorough clinical evaluation, involving a patient’s detailed history, and specialized tests including biochemical analysis [e.g., elevated serum creatine kinase (CK), a marker of muscle necrosis [14]] and molecular genetic testing for dystrophin mutations. Various factors, such as proteins [e.g., lactate dehydrogenase (LDH)], lipids and metabolites (e.g., fatty acids, carnosine, taurine, and creatine), microRNAs (e.g., miR-1, miR-31, miR-133a, and miR-206), and genomic factors (e.g., latent TGFβ-binding protein 4—LTBP4 genotype) (reviewed in: [15]), may be helpful in DMD diagnosis; however, they are not specific. The potential applicability of serum levels of matrix metalloproteinase 9 (MMP-9), myostatin (GDF-8), and follistatin as non-invasive biomarkers was also suggested [16]. The age of onset and the rate of decline may vary among DMD boys; however, the first signs of motor impairment and difficulty in walking are observed between 1 and 3 years of age. In most cases, rapid disease progression and muscle-weakening occur between the ages of 10 and 14, and by age 20, affected individuals already begin to suffer from respiratory and cardiac failure, which leads to death in the 2nd or 3rd decades of their life [17].

**Fig. 2:**
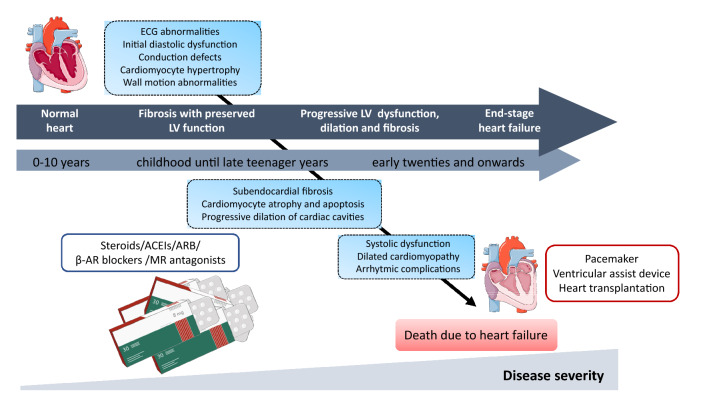
Progressive cardiovascular dysfunctions in patients with DMD. In early childhood, a normal ventricular function is detected, which progresses to end-stage heart failure demonstrated by systolic dysfunction and dilated cardiomyopathy. Steroids, angiotensin-converting enzyme inhibitors (ACE inhibitors), angiotensin II receptor blockers (ARB), beta-adrenergic receptor blockers (β-AR blockers), or mineralocorticoid receptor (MR) antagonists may be used to treat cardiac problems. Various implantable devices may be used as mechanical support as the disease becomes more severe. *LV* left ventricular

Respiratory muscle weakness and decreased pulmonary function with a high incidence of respiratory infections are serious problems in DMD patients. The common standards of care include frequent respiratory function assessments as well as the use of respiratory assist devices and non-invasive ventilation (NIV), which decreases the risk of hypoventilation events and improves the quality of life. However, improving the management of the devastating consequences of skeletal muscle and pulmonary dysfunctions and the prolongation of life expectancy may lead to the appearance of cardiac problems associated with dystrophinopathies (Fig. 2). Of note, the incidence of dilated cardiomyopathy, characterized by left-ventricular (LV) dilation and decrease of the wall diameter resulting in a reduction in the cardiac ejection fraction [18], increases with age and more than 90% of young DMD men over 18 demonstrate evidence of cardiac dysfunction [19]. Additional cardiac complications including conduction and electrocardiogram (ECG) abnormalities like atrial and ventricular tachycardias as well as atrial arrhythmias may contribute significantly to DMD patients’ morbidity and mortality (references in [18–21]).

In cardiomyocytes, dystrophin exerts the same functions as in skeletal muscle cells; therefore, a lack of dystrophin results in increased cardiomyocyte structural vulnerability, membrane instability, disruption in Ca^2+^ homeostasis, augmented reactive oxygen species (ROS) production, and mitochondrial dysfunction [21]. In DMD heart, not only dystrophin-deficient cardiomyocytes exert impairment in their beating capacities—as dystrophin is expressed also in endothelial cells, vascular smooth muscle cells, and fibroblasts [22–25], and perturbations in the functioning of all those cells are responsible for cardiac complications. Of note, the functioning of dystrophic blood vessels is improper [22] causing decreased vascularisation of the muscle [23, 24].

Heart problems in DMD patients can be undetected without detailed examination [26, 27]. Many individuals have no classic symptoms of heart failure or they are unrecognized properly, as DMD individuals are mostly wheelchair bound and do not perform increased cardiac workload. Unfortunately, it leads to a significant delay in their proper evaluation and late initiation of pharmacological treatment [28]. However, to prevent the early onset of heart failure, it is suggested to start treatment before ventricular dysfunction is detected. In patients with end-stage heart failure, mechanical cardiac support by the use of various implantable devices including left-ventricular assist devices (LVADs) or pacemakers may be helpful, although their usage creates ethical questions [18, 26, 29–31]. Additionally, heart transplantation might be a last resort, but the possible postoperative complications, such as bleeding, arrhythmias, stroke, respiratory failure, and others, have to be considered [18].

**Fig. 3:**
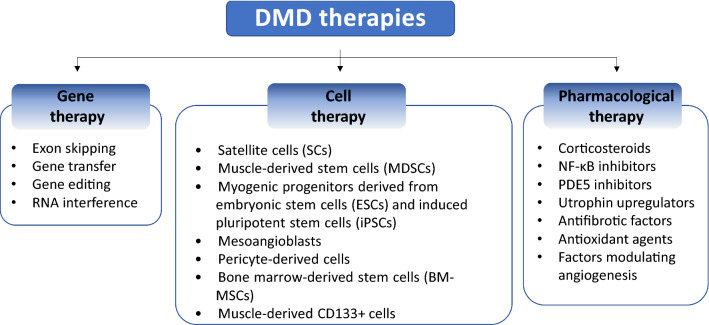
Possible therapies in DMD treatment. Current strategies rely on gene, cell, and pharmacological-based therapeutic approaches. See details in the text

DMD remains an incurable disease. Even though it was first mentioned in the early nineteenth century by Italian physicians Conte and Gioja and described in detail by French neurologist, Guillaume Duchenne in the 1860s followed by many further studies [32], there is still no effective treatment available for all DMD patients. Although novel therapeutics applying gene therapy-based techniques have experienced a dramatic advance in the past 15 years, also in the context of DMD, only a small number of patients are amenable for treatment with mutation-specific drugs (described later). Therefore, there is a constant need to investigate novel approaches aimed at modulating the severity of the disease, at best. Discoveries in recent years have brought new strategies, and in this review, we will focus on the critical summary of the selected examples of the gene, cell, and pharmacological therapies suggested to be beneficial in experimental DMD models (Fig. 3). Currently, around 280 clinical trials with different statuses (131 completed; 29 terminated; 43 recruiting; 10 not yet recruiting; 10 enrolling by invitation; 19 active, not recruiting; 2 suspended; 7 withdrawn; 25 unknown status) are registered at www.clinicaltrials.gov. Selected trials are summarized in Table 1 and described in the ensuing chapters. However, it has to be underlined that registration of the trial at this database does not automatically indicate that the proposed treatment is safe and is supported by the strong scientific evidence.

**Table 1:** Selected completed or ongoing clinical trials for DMD

Study title	Tested compound	Drug route	Phase	Additional information	NCT number	Recruitment status	Company/sponsor
**Gene-based therapies**							
*Micro- and minidystrophin overexpression*							
Systemic Gene Delivery Clinical Trial for Duchenne Muscular Dystrophy	rAAVrh74.MHCK7	IV	1	A single-dose-controlled trial using AAV9 based gene therapy (rAAVrh74.MHCK7.microdystrophin)	*NCT03375164*	Active, not recruiting	Sarepta Therapeutics
A Randomized, Double-blind, Placebo-controlled Study of SRP-9001 for Duchenne Muscular Dystrophy (DMD)	SRP-9001	IV	2	A 48-week systemic, gene-delivery clinical trial using SRP-9001 (AAVrh74.MHCK7.microdystrophin)	*NCT03769116*	Active, not recruiting	Sarepta Therapeutics
A Study to Evaluate the Safety and Tolerability of PF-06939926 gene therapy in Duchenne muscular dystrophy	PF-06939926	IV	1	A safety and tolerability study with AAV9 vector carrying a truncated human dystrophin gene (minidystrophin) under the control of a human muscle-specific promoter	*NCT03362502*	Recruiting	Pfizer
Microdystrophin gene transfer study in adolescents and children with DMD (IGNITE DMD)	SGT-001	IV	1	A randomized, controlled, open-label, single-ascending dose study with AAV9 vector containing the muscle-specific promoter and microdystrophin construct	*NCT03368742*	Suspended (Clinical Hold)	Solid Biosciences
*Exon skipping approach*							
Safety study of eteplirsen to treat early stage Duchenne muscular dystrophy	Eteplirsen (EXONDYS 51)	IV	2	A 96-week-long study performed on 20 DMD patients amenable to exon 51 skipping	*NCT02420379*	Completed	Sarepta Therapeutics
Dose-titration and open-label extension study of SRP-4045 in advanced-stage Duchenne muscular dystrophy (DMD) patients	SRP-4045	IV	1	A first-in-human dose-titration and open-label extension study to assess safety, tolerability, and PK of SRP-4045 in advanced-stage DMD patients with deletions amenable to exon 45 skipping	*NCT02530905*	Completed	Sarepta Therapeutics
Phase I/II study of SRP-4053 in DMD patients	SRP-4053	IV	1/2	A first-in-human, multiple-dose 2-part study to assess the safety, tolerability, efficacy, and PK of SRP-4053 in patients amenable to exon 53 skipping	*NCT02310906*	Completed	Sarepta therapeutics
Study of SRP-4045 and SRP-4053 in DMD patients (ESSENCE)	SRP-4053/SRP-4045	IV	3	A double-blind, placebo-controlled study to evaluate the efficacy and safety of SRP-4045 and SRP-4053 in patients with out-of-frame deletion mutations amenable to exon 45 or 53 skipping	*NCT02500381*	Recruiting	Sarepta Therapeutics
A 48-week, open label, study to evaluate the efficacy and safety of casimersen, eteplirsen and golodirsen in subjects with Duchenne muscular dystrophy carrying eligible DMD duplications	Casimersen Eteplirsen Golodirsen	IV	2	A 1-year-study in DMD subjects with duplication mutations amenable to treatment by exon 45, 51 or exon 53 skipping	*NCT04179409*	Enrolling by invitation	Sarepta Therapeutics
An extension study to evaluate casimersen or golodirsen in patients with Duchenne muscular dystrophy	Casimersen (SRP-4045) Golodirsen (SRP-4053)	IV	3	Long-term (up to 144 weeks) trial in patients amenable to exon 45 or 53 skipping	*NCT03532542*	Enrolling by invitation	Sarepta Therapeutics
Safety and dose finding study of NS-065/NCNP-01 in boys with Duchenne muscular dystrophy (DMD)	Viltolarsen (NS-065/NCNP-01)	IV	2	A study to evaluate the safety of a high (80 mg/kg) and low (40 mg/kg) dose of NS-065/NCNP-01 in DMD patients amenable to exon 53 skipping	*NCT02740972*	Completed	NS Pharma
Extension study of NS-065/NCNP-01 in boys with Duchenne muscular dystrophy (DMD)	Viltolarsen (NS-065/NCNP-01)	IV	2	An open-label, extension study of NS-065/NCNP-01 administered intravenously once weekly for an additional 144 weeks to boys with DMD who complete study NS-065/NCNP-01-201	*NCT03167255*	Active, not recruiting	NS Pharma
Study of DS-5141b in patients with Duchenne muscular dystrophy	DS-5141b	SC	1/2	A study to evaluate the safety, tolerability, efficacy, and PK profile of DS-5141b in DMD patients amenable to exon 45 skipping	*NCT02667483*	Active, not recruiting	Daiichi Sankyo
*Readthrough therapy*							
Study of ataluren in ≥ 2 to < 5-year-old males with Duchenne muscular dystrophy	Ataluren	PO	2	A phase 2, multiple-dose, open-label study evaluating the safety, PK, and PD of ataluren in nonsense mutations DMD patients	*NCT02819557*	Completed	PTC Therapeutics
Safety and efficacy study of PTC124 in Duchenne muscular dystrophy	Ataluren	PO	2	A phase 2 study to understand whether ataluren can safely increase functional dystrophin protein in the muscles of patients with DMD due to a nonsense mutation	*NCT00264888*	Completed	PTC Therapeutics
Phase 2B study of PTC124 (ataluren) in Duchenne/Becker muscular dystrophy (DMD/BMD)	Ataluren	PO	2	A Phase 2b, multicenter, randomized, double-blind, placebo-controlled, dose-ranging, efficacy, and safety study, designed to document the clinical benefit of ataluren when administered as therapy of patients with DMD/BMD	*NCT00592553*	Completed	PTC Therapeutics
Phase 3 study of ataluren in patients with nonsense mutation Duchenne muscular dystrophy (ACT DMD)	Ataluren	PO	3	A Phase 3, multicenter, randomized, double-blind, placebo-controlled study to determine the efficacy and safety of 10, 10, 20 mg/kg ataluren given 3 times/day for 48 weeks	*NCT01826487*	Completed	PTC Therapeutics
Registry of translarna (Ataluren) in nonsense mutation Duchenne muscular dystrophy (nmDMD)	Ataluren	PO	4	A post-approval safety study (PASS), per the Pharmacovigilance Risk Assessment Committee (PRAC) of the European Medicines Agency (EMA), to gather data on Translarna (ataluren) safety, effectiveness, and prescription patterns in routine clinical practice	*NCT02369731*	Recruiting	PTC Therapeutics
Phase II study of NPC-14 (Arbekacin Sulfate) to explore safety, tolerability, and efficacy in Duchenne muscular dystrophy (NORTH POLE DMD)	NPC-14 (Arbekacin Sulfate)	IV	2	A randomized, double-blind, placebo-controlled study with NPC-14 for 36 weeks in 21 ambulant DMD patients with nonsense mutation	*NCT01918384*	Unknown	Kobe University
6-month study of gentamicin in Duchenne muscular dystrophy with stop codons	Gentamicin	IV	1	A study to determine the safety of gentamicin in DMD boys who have stop codon mutations	*NCT00451074*	Completed	Nationwide Children's Hospital
*Other gene overexpression*							
Gene transfer clinical trial to deliver rAAVrh74.MCK.GALGT2 for Duchenne muscular dystrophy	rAAVrh74.MCK.GALGT2	ILI	1/2	An open-label, dose-escalation trial with vector delivery via the femoral artery to the muscles of both legs of DMD subjects	*NCT03333590*	Active, not recruiting	Kevin Flanigan
Follistatin gene transfer to patients with becker muscular dystrophy and sporadic inclusion body myositis	rAAV1.CMV.huFollistatin344	IM	1	A safety study to evaluate the effect of follistatin gene therapy in 3 different doses	*NCT01519349*	Completed	Nationwide Children's Hospital
Clinical intramuscular gene transfer of rAAV1.CMV.huFollistatin344 trial to patients with Duchenne Muscular Dystrophy	rAAV1.CMV.huFollistatin344	IM	1/2	Intramuscular gene transfer of follistatin at a total dose of 2.4 × 10^12^ vg/kg (1.2 × 10^12^ vg/kg/limb) to six DMD patients	*NCT02354781*	Completed	Jerry R. Mendell
**Cell therapies**							
HOPE-Duchenne (Halt cardiomyOPathy progrEssion in Duchenne) (HOPE)*	Allogeneic Cardiosphere-Derived Cells (CAP-1002)	IC	1/2	A study with the infusion of CAP-1002 in three coronary arteries supplying the three major cardiac territories of the left ventricle of the heart (anterior, lateral, inferior/posterior) (note: the study was not double-blind; the existence of cardiac stem cells has been falsified; there is a concern on the efficacy of the mode of delivery of the cardiospheres; limitations recognized by the authors are listed in the paper [175]	*NCT02485938*	Completed	Capricor Inc
Bone marrow-derived autologous stem cells for the treatment of Duchenne muscular dystrophy**	Bone marrow-derived stem cells		1/2	Transplantation of purified autologous bone marrow-derived stem cells (note: bone marrow-derived stem cells do not differentiate into the muscles; autologous cells still have DMD mutation)	*NCT03067831*	Recruiting	Stem Cells Arabia
Safety and efficacy of umbilical cord mesenchymal stem cell therapy for patients with Duchenne muscular dystrophy**	Human umbilical cord mesenchymal stem cells		1/2	Participants will be given rehabilitation therapy plus human umbilical cord mesenchymal stem cells transplantation with 1-year follow-up (note: lack of valid evidence of the so-called MSC to differentiate into the muscles; concerns on immune reactions)	*NCT01610440*	Unknown	Shenzhen Beike BioTechnology
**Pharmacological therapies**							
*Utrophin upregulation*							
Proof of concept study to assess activity and safety of SMT C1100 (Ezutromid) in boys with Duchenne muscular dystrophy	SMT C1100 (Ezutromid)	PO	2	The study to evaluate the activity and safety of utrophin modulation was terminated due to a lack of efficacy in cohorts 1 and 2	*NCT02858362*	Terminated	Summit Therapeutics
*Cardiac therapy*							
Plus epicatechin Duchenne muscular dystrophy in non-ambulatory adolescents	Epicatechin	PO	1/2	A pilot study on 15 non-ambulatory DMD children at least 8 years of age with preclinical cardiomyopathy	*NCT02964377*	Completed	Cardero Therapeutics
Therapeutic potential for aldosterone inhibition in Duchenne muscular dystrophy	Spironolactone *vs* eplerenone	PO	3	The study is to demonstrate non-inferiority of spironolactone vs eplerenone in preserving cardiac and pulmonary function in patients with preserved LV ejection fraction	*NCT02354352*	Completed	Ohio State University
Nebivolol for the prevention of left ventricular systolic dysfunction in patients with Duchenne muscular dystrophy (NEBIDYS)	Nebivolol	PO	3	The objective is to determine whether nebivolol, a beta-blocker, can prevent the development of heart disease in 10 to 15-year-old DMD patients	*NCT01648634*	Active, not recruiting	Assistance Publique–Hôpitaux de Paris
Duchenne muscular dystrophy heart study (DMD-HS)	Observational study	–	–	A retrospective cohort study on genetically proven DMD patients diagnosed from 01.1993–03.2020 to assess the extent of dilated cardiomyopathy	*NCT03443115*	Unknown	Association Monégasque contre les Myopathies
*Muscle ischemia*							
PDE inhibitors in DMD study (acute dosing study)	Sildenafil Tadalafil	PO	1	12 DMD subjects were given both open-label sildenafil initially and then tadalafil to assess their effect on skeletal and cardiac endpoints	*NCT01580501*	Completed	Cedars-Sinai Medical Center
A study of tadalafil for Duchenne muscular dystrophy	Tadalafil	PO	3	Long-term evaluation of tadalafil treatment on ~ 300 individuals was terminated for the lack of efficacy	*NCT01865084*	Terminated	Eli Lilly and Company
*Myostatin inhibition*							
Clinical trial to evaluate the efficacy, safety, and tolerability of RO7239361 in ambulatory boys with Duchenne muscular dystrophy	RO7239361 (BMS-986089)	SC	2/3	A multicenter, randomized, double-blind, placebo-controlled study to assess the efficacy, safety, and tolerability of two different weekly doses of the anti-myostatin drug	*NCT03039686*	Terminated	Hoffmann-La Roche
An open-label extension study to evaluate safety of PF-06252616 in boys with Duchenne muscular dystrophy	PF-06252616	IV	2	Terminated on 30 Aug 2018 due to the lack of efficacy	*NCT02907619*	Terminated	Pfizer
Study evaluating MYO-029 in adult muscular dystrophy	MYO-029	IV	2	A study to assess the safety of MYO-029 in adult patients with muscular dystrophy	*NCT00104078*	Completed	Wyeth/Pfizer
Study of ACE-031 in subjects with Duchenne muscular dystrophy	ACE-031	SC	2	A study with ACE-031, a soluble form of the human activin receptor type IIB, was terminated based on safety data	*NCT01099761*	Terminated	Acceleron Pharma
Extension study of ACE-031 in subjects with Duchenne muscular dystrophy	ACE-031	SC	2	The long-term safety and tolerability of ACE-031 administration in subjects who participated in study NCT01099761 was terminated based on preliminary safety data	*NCT01239758*	Terminated	Acceleron Pharma
*Corticosteroids*							
A pharmacokinetic study of oral deflazacort in children and adolescent subjects with duchenne muscular dystrophy	Deflazacort	PO	1	Study to characterize the 8 day dosing of oral deflazacort in pediatric and adolescents subjects	*NCT02251600*	Completed	PTC Therapeutics
An open-label, long-term extension study to evaluate the safety and tolerability deflazacort	Deflazacort	PO	1	Further evaluation of the safety and possible effects after deflazacort administration	*NCT02295748*	Completed	PTC Therapeutics
High-dose prednisone in Duchenne muscular dystrophy	Prednisone	PO	3	A study to check if a high-dose weekly course of prednisone therapy is safer and at least as effective as daily dose therapy	*NCT00110669*	Completed	Cooperative International Neuromuscular Research Group
Finding the optimum regimen for Duchenne muscular dystrophy (FOR DMD)	Prednisone Deflazacort	PO	3	The aim is to compare three ways of corticosteroids administration to DMD boys	*NCT01603407*	Completed	University of Rochester
A study to assess vamorolone in boys with Duchenne muscular dystrophy (DMD)	Vamorolone	PO	2	The aim was to evaluate the safety and tolerability of four different doses of vamorolone administered orally in DMD boys ages 4–7 years	*NCT02760264*	Completed	ReveraGen BioPharma
An extension study to assess vamorolone in boys with Duchenne muscular dystrophy (DMD)	Vamorolone	PO	2	Continuation of the study #NCT02760264 with 24 weeks administration of the drug	*NCT02760277*	Completed	ReveraGen BioPharma
Long-term extension study to assess vamorolone in boys with Duchenne muscular dystrophy (DMD)	Vamorolone	PO	2	Continuation of the study #NCT02760277 with 24 months administration of the drug	*NCT03038399*	Active, not recruiting	ReveraGen BioPharma
*Anti-inflammatory factors*							
A two-part study to assess the safety and tolerability, pk, effects on histology and some clinical parameters of givinostat in ambulant children with DMD	Givinostat	PO	1/2	The safety, tolerability, and PK of histone deacetylase inhibitor treatment for a maximum 12 months	*NCT01761292*	Completed	Italfarmaco
Givinostat in Duchenne's muscular dystrophy long-term safety and tolerability study	Givinostat	PO	2/3	An extension of the previous study with HDAC inhibitor (NCT01761292)	*NCT03373968*	Recruiting	Italfarmaco
Clinical study to evaluate the efficacy and safety of givinostat in ambulant patients with Duchenne muscular dystrophy	Givinostat	PO	3	A randomized, double-blind, parallel-group, placebo-controlled study planned to be performed on a total of 213 subjects	*NCT02851797*	Recruiting	Italfarmaco
Phase 1/2 study in boys with Duchenne muscular dystrophy (MoveDMD^®^)	Edasalonexent (CAT-1004)	PO	1/2	A 3-part, multi-site study to evaluate the safety, efficacy, PK, and PD of small-molecule targeted to inhibit activated NF-κB in pediatric DMD patients (≥ 4 to < 8 years of age)	*NCT02439216*	Completed	Catabasis Pharmaceuticals
A study of TAS-205 for Duchenne muscular dystrophy	TAS-205	PO	1	To evaluate the safety and PK of hematopoietic-type prostaglandin D synthase (HPGDS) inhibitor	*NCT02246478*	Completed	Taiho Pharmaceutical
A phase IIa study of TAS-205 for Duchenne muscular dystrophy	TAS-205	PO	2	To check the efficacy after 24-week repeated oral doses of TAS-205	*NCT02752048*	Completed	Taiho Pharmaceutical
*Anti-fibrotic agents*							
Trial of Pamrevlumab (FG-3019), in non-ambulatory subjects with Duchenne muscular dystrophy (DMD)	Pamrevlumab (FG-3019)	IV	2	To assess the effectiveness of a monoclonal antibody to Connective Tissue Growth Factor in a study with an intravenous infusion of FG-3019 every 2 weeks by for up to 156 weeks	*NCT02606136*	Active, not recruiting	FibroGen

Various gene, cell, and pharmacological therapies are shown (based on: clinicaltrials.gov). Despite the common belief, the registration of the trials in clinicaltrials.gov does not undergo a stringent peer-review process, and some of the trials posted there cannot be considered as having a strong biomedical rationale [276, 277]. This, in particular, concerns many trials based on poorly defined cells, often named “stem“ cells without sufficient proof [278]. We have carefully analysed the database content in regard to DMD taking into consideration the above limitations. Please refer to the manuscript text critically discussing the rationale of some cell-therapy approaches in DMD. In our opinion, there is not sufficient justification for the trials marked with * (or **—even less justified) in the table above

*ILI* intravascular limb infusion, *IV* intravenous infusion, *SC* subcutaneous injection, *PO* oral administration, *PK* pharmacokinetics, *PD* pharmacodynamics, *NCT* number: ClinicalTrials.gov identifier

## Gene-based therapies

DMD is an attractive candidate for gene therapy, as it arises from single-gene mutations. It is believed that gene therapy will provide great opportunities for patients; however, so far, the approach has been highly challenging [33]. The optimal way of gene delivery, the appropriate (minimal) level of dystrophin expression, needed to stop disease progression and the prevention of immune reaction not only in response to gene therapy itself but also following the reintroduction of a gene whose product may be recognized as foreign by the immune system of DMD patients [34] are the most crucial factors which need to be optimized. As all dystrophic muscles lack dystrophin, efficient gene therapy should allow the expression of a new dystrophin gene not only in limb muscles but also in the diaphragm and the heart. The estimation of how much dystrophin is required to speculate about the beneficial effects of the therapy is very challenging and questionable as various methods, measuring different outcomes, have been used. It was shown that, in *mdx* mice, as little as 20% of the wild-type dystrophin level was effective in the prevention of disease progression when the creatine kinase level and fiber degeneration were assessed, but for cardiomyopathy treatment, this level should exceed 50% [35, 36]. In humans, it was speculated that around 30% of the wild-type dystrophin level is efficient [37]. However, the necessary amount might be dependent on the disease’s progression and individual condition of the patient. A recent report on dystrophin quantification listed several factors that may influence its level, including the structure and functionality of the new protein and the distribution of the dystrophin in muscle fibers. It has to be underlined that calculation of the amount of dystrophin may be greatly affected by the method used (and may even be misleading)—western blot analysis does not show if there is any dystrophin in all fibers, or higher dystrophin levels in some fibers, whereas immunofluorescence gives information about localization, but is not a quantitative method [38].

### Minidystrophin and microdystrophin overexpression using AAVs vectors

DMD is caused by recessive and monogenic genetic mutations, mostly large deletions, duplications, rearrangements, and point mutations [2]; therefore, therapeutic strategies aimed at the correction or improvement of muscle function by exogenous delivery of functionally engineered dystrophin gene constructs or augmentation of the endogenous locus have been broadly studied. Although different viral vectors can be used to deliver genes to muscle fibers, the recombinant adeno-associated virus (AAV)-based vectors are the most suitable in *DMD* gene therapies. The major advantages of AAV vectors include the ability to transduce non-dividing cells and possibility to provide the long-term expression of the delivered transgenes [39]. From several serotypes of AAV, serotypes 1, 6, 8, and 9 are extremely useful for DMD therapy as they exhibit a potent tropism for striated muscles [40]. For cardiac gene delivery by systemic vector administration, AAV9 was shown to be superior to other serotypes, including AAV8 [41].

**Fig. 4:**
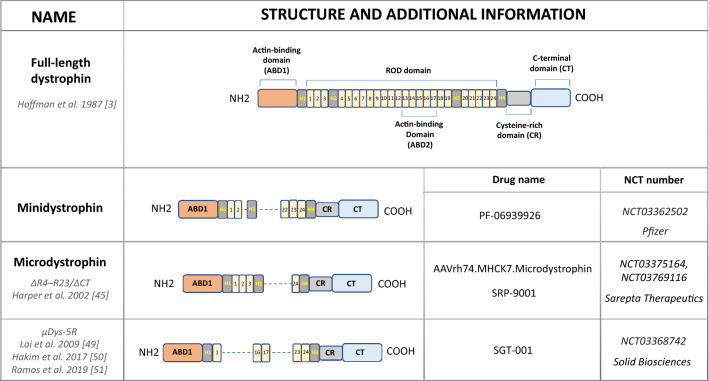
A comparison of full-length dystrophin and truncated forms—minidystrophin and microdystrophin currently in use in clinical trials. The differences in the structure and additional information, including the number of the clinical trial and the commercial name of the drug are shown. Domains within dystrophin are abbreviated as follows: *ABD* actin-binding domain, *1–24* spectrin-like repeats, *H* hinge domains, *CR* cysteine-rich domain, *CT* carboxy-terminal domain

The restoration of the dystrophin expression by gene transfer of full dystrophin coding sequence has been tested experimentally and in some clinical trials [42]. This is very challenging due to the size of the dystrophin gene, as the total dystrophin cDNA, counting 14 kb nucleotides cannot be packed in the majority of the available vectors. AAV vectors have a limited carrying capacity as a 5 kb genome is considered to be the upper limit for a single AAV virion. Dystrophin is built of four major structural domains (Fig. 4): an N-terminal actin-binding domain (ABD1), and a central rod domain (containing ABD2) composed of 24 spectrin-like repeats and 4 hinge domains (H1–H4). In the last H4 domain, WW domain responsible for binding to part of the DGC is present. Finally, a cysteine-rich domain (CR) followed by a distal C-terminal domain that interacts with members of DGC at the sarcolemmal membrane is found in the full version of the protein. Of note, it has been found that some internal parts of the dystrophin sequence are dispensable for protein functioning and a series of rod-truncated and the C-terminal domain lacking dystrophin genes were proposed to be used instead of the full protein [33, 42, 43]. This discovery has been possible thanks to elucidating the nature of *DMD* gene mutations in Becker muscular dystrophy (BMD), the milder and much rarer form of the dystrophin-dependent disease, in which the mutations result in loss of some exons, but do not abolish the dystrophin expression, as is the case in DMD [44]. Accordingly, it has been found that an artificially truncated version of dystrophin, lacking the internal part, like in BMD mutation, can be packed even in the small AAV vectors, creating the chance for the in vivo gene therapy strategies [18, 42]. Therefore, the functional but internally deleted “mini”- dystrophin [45] and “micro”- dystrophin [46] constructs to facilitate gene transfer have been established. Of note, around 40 constructs of microdystrophin have been tested in animal models (reviewed in: [47]). However, the clinical efficacy of so far performed studies is far from expected, and this is, among others, due to the large mass of the muscles to be transduced, difficulty in transducing the diaphragm and particularly the heart. One of the first clinical trials with microdystrophin [2 × 10^10^ vector genomes per kilogram of body weight (vg/kg) or 1 × 10^11^ vg/kg injected to the biceps] in six patients with DMD conducted in 2006 by Mendell et al. at the Nationwide Children’s Hospital in Columbus, Ohio USA [34] showed a very low level of microdystrophin expression (~ 3–4 positive myofibers in one low-dose patient, and 1 positive myofiber detected in one high-dose patient at day 42). These results were far from the expected and the expression of the product was not sufficient for efficient therapy. However, recent paper summarizing the results of 1 year, nonrandomized-controlled trial (called Study-101, NCT03375164) with microdystrophin gene therapy using AAVrh74 vector, isolated from lymph nodes of rhesus monkeys and sharing 93% amino acid identity to AAV8 (SRP-9001; AAVrh74.MHCK7) in four young DMD patients [48], showed increased levels of dystrophin by 81.2% in the muscles without signs of severe adverse effects. Currently, a phase 2 randomized, double-blind, placebo-controlled trial with SRP-9001 known as Study-102 (NCT03769116) is ongoing (Table 1).

It has to be emphasised that in this and the other studies with viral overexpression of microdystrophin, the patients were simultaneously treated with corticosteroids to prevent an immune response against the viral vector. As the patients received a high dose for the first 30 days, followed by their standard-of-care corticosteroid dose, it cannot be excluded that the observed benefits may be the result of steroid treatment as well. Similarly, Pfizer has conducted a phase 1b trial (NCT03362502) with AAV9 carrying a minidystrophin gene under the control of a human muscle-specific promoter (PF-06939926). This study, aiming to assess the safety and tolerability of this approach is ongoing; however, in 2020, Pfizer is going to perform a second, randomized, placebo-controlled phase 3 study to further test PF-06939926. Similarly, Solid Biosciences, created SGT-001 (NCT03368742), AAV9 vector containing the muscle-specific promoter and microdystrophin. This construct, known also as μDys5R microdystrophin, carries the R16/17 nNOS-binding domain and was shown to efficiently restore nNOS localization in dystrophic animals [49–51]. The trial has started in December 2017, and the patients, after receiving a single intravenous infusion of SGT-001, have been monitored for approximately 2 years. Preliminary results indicated that low levels of microdystrophin were present in the muscles of the treated patients, and no serious adverse events were reported (https://musculardystrophynews.com/). Of note, recently Pfizer has announced that in addition to mild adverse events (AEs) including vomiting, nausea, decreased appetite, and pyrexia, in four out of nine patients, serious AEs were described. In one patient, strong immune reaction occurred [atypical hemolytic uremic syndrome (aHUS)-like complement activation, which required hemodialysis and treatment with the kidney drug—eculizumab] [52]. Similarly, in the trial performed by Solid Biosciences, a 7-year-old patient was hospitalized as decreased red blood cell count, acute kidney injury, and cardio-pulmonary complications were noted after receiving a higher dose of SGT-001. As a consequence, in November 2019, the trial was placed on clinical hold, and even after sending an explanation by the company in April 2020, the Food and Drug Administration (FDA) responded by requesting further data and analyses relating to the manufacturing process (www.solidbio.com, [53]).

### Utrophin replacement

One concern of dystrophin replacement therapy is related to the immune reaction in response to the newly generated dystrophin protein [34, 54]. Immunosuppressant drugs may, therefore, be necessary. However, another option is based on the upregulation of utrophin, the related protein that disappears in a majority of human muscles after birth. Utrophin is a structural (~ 80% of homology) and functional autosomal paralogue of dystrophin [55], expressed in developing muscles at the sarcolemma [56] and progressively replaced by dystrophin [57]. In adults, it is mostly expressed in the lung, kidney, liver, and spleen [58], neuromuscular junction, and myotendinous junction in mature muscles [59] and in the sarcolemma in regenerating myofibers [60]. Therefore, it was speculated that it might have activities redundant to dystrophin and was tested to compensate for the dystrophin deficiency. This idea is also supported by the observation that double dystrophin-utrophin knock-out mice demonstrated more severe muscle weakness and cardiac abnormalities with reduced life expectancy than *mdx* mice being dystrophin-only mutant [61, 62]. On the other hand, the upregulation of utrophin in *mdx* mice (by crossing transgenic mice expressing the full-length utrophin protein in skeletal muscle with dystrophin-deficient *mdx* mice) positively affected muscle morphology, fiber regeneration, and mechanical properties [63]. The mechanism exerted by utrophin may also rely on the positive effects on mitochondrial dysfunctions observed during dystrophy progression. Using dystrophin-deficient *mdx*/utrophin overexpressing Fiona (*mdx/*Fiona) transgenic mice, Kennedy et al. [64] have demonstrated that high levels of utrophin ameliorate the aberrant structure and localization of mitochondria as well as reduce oxidative stress.

Still, new possibilities to overexpress utrophin are suggested. Utrophin is repressed by several microRNAs, including let-7c and the strategy of applying an oligonucleotide, able to anneal to the utrophin 3′UTR and prevent let-7c binding, thereby upregulating utrophin expression was tested [65]. Mishra et al. have used oligonucleotide composed of 2′-*O*-methyl modified bases on a phosphorothioate backbone (let7-SBOs) to evaluate its effectiveness in *mdx* mice. The intraperitoneal (i.p.) injection of let7-SBOs led to increased expression of utrophin in diaphragm, gastrocnemius, and tibialis anterior muscles of 2-month-old *mdx* mice after 1 month of treatment. The delivery of let7-SBOs was also able to improve the dystrophic phenotype in vivo as assessed by morphological and physiological properties of muscles (e.g., muscle weight, muscle damage, inflammatory cell infiltration, and fibrosis as well as specific force) [65].

An important advantage of utrophin delivery might be a minimal risk of the immune response which was reported after a high dose of the AAV-minidystrophin gene, for example, in the canine model of DMD and GRMD dogs [66]. Song et al. [67] performed a series of experiments aiming to compare the effectiveness of miniaturized utrophin, delivered by AAVs to similarly constructed microdystrophin. Not only did it prevent muscle disease in newborn *mdx* mice, but it was evidenced that there was a lack of immune response in neonatal GRMD dogs and amelioration of dystrophic symptoms without inducing T-cell responses in adult dogs deficient in the entire dystrophin [67].

Although utrophin exerts beneficial effects and is used to compensate for dystrophin deficiency, it must be stated that both proteins differ in some functions. One of the most important may be that utrophin is not able to prevent functional ischemia during muscle contraction, the effect exerted by dystrophin as a consequence of nitric oxide synthase (nNOS) anchoring to sarcolemma [49, 68]. Dystrophin binds microtubules to form a rectilinear lattice beneath the sarcolemma, whereas utrophin cannot [69]. Therefore, utrophin therapy is not able to correct subsarcolemmal microtubule lattice disorganization and was not effective when physical activity after mild exercise or torque production after in vivo eccentric contraction in dystrophin-deficient skeletal muscle was evaluated [69].

As those differences may have important clinical consequences, a combination of utrophin upregulation with dystrophin-based therapies for DMD has to be considered. Guiraud et al. [70] using various strains of mice differing in the expression of dystrophin and utrophin as well as by combining utrophin overexpression and dystrophin restoration with exon skipping (see “Exon skipping approach”) in dystrophic muscle have demonstrated the additive benefits of such treatment over mono-therapies. It led the authors to suggest that utrophin overexpression may be particularly beneficial in BMD patients who express a low level of dystrophin [70].

Not only is gene therapy under evaluation, but many pharmacological ways to upregulate utrophin are also being considered (see “The upregulation of utrophin by drug therapy is a plausible therapeutic approach in the treatment of DMD”).

### Exon skipping approach

Another form of genetic therapy relies on the application of antisense oligonucleotides (ASOs), which, by binding to the exon/intron boundary or by targeting intra-exonic regions, can cause the exon skipping, and restoring the reading frame, leading to the expression of a truncated but functional protein [71]. As discussed above, this approach relies on the observation of some BMD patients, carrying in-frame exonic deletions, giving a dystrophin product that is truncated but highly functional [72].

**Fig. 5:**
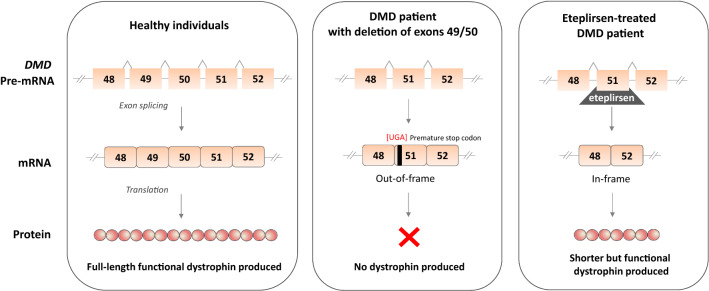
Mode of eteplirsen action. In healthy individuals, dystrophin is produced, whereas in a DMD patient, deletion spanning exons 49 and 50 create out-of-frame frameshift that introduces a premature stop codon and result in a lack of dystrophin production. In such patients, eteplirsen, the exon skipping ASO targeting exon 51 of the *DMD* gene can be used. After hybridization to pre-mRNA, it affects the splicing machinery to skip exon 51 from the mature mRNA transcript. This converts the out-of-frame into the in-frame transcript coding for a shorter but functional protein

Chemically, phosphorodiamidate morpholino oligomers (PMOs), 2-O-methyl-modified RNA, and tricycloDNA antisense are used, among other modifications [73], for such a purpose. Several compounds were tested in various DMD animal models [74] and even demonstrated satisfactory results in phase 2 clinical trials [75, 76] (Table 1). Eteplirsen (or EXONDYS 51; Sarepta Therapeutics) is a PMO skipping the exon 51 of *DMD* gene and allowing to restore the dystrophin expression lost by the deletion of exon 49/50 (Fig. 5). Of note, skipping the exon 51 may be beneficial for a larger group of DMD patients with deletions ending at exon 50 or starting at exon 52 (e.g.,, 45–50, 47–50, 48–50, 49–50, 50, 52, and 52–63) [77] and it is estimated to be used in approximately 13–14% of DMD boys [14, 78].

Unfortunately, it is still not clear whether the effectiveness of eteplirsen is as it was initially claimed and quite big discrepancies in the level of dystrophin protein detected in patients undergoing this therapy were noted (mostly being the result of various methods used to assess the dystrophin level) [78]. An analysis of the results of clinical trials with eteplirsen (NCT01396239/NCT01540409) revealed that after 180 weeks of treatment, western blot-based quantification showed 0.93% of dystrophin levels observed in healthy individuals, whereas dystrophin-positive fiber counts assessed by IHC were detected at 17.4% on average.

In addition to the controversial assessment of an increase in dystrophin in muscle biopsy specimens, there were many additional doubts from the DMD community about the way which the drug was developed, the initial trial performed on the small size group, and, finally, that there were no obvious advantages in the functional test, the 6-min walk distance (6MWD) test capacity, between patients who received eteplirsen and those initially given a placebo [79]. Anyway, the drug was conditionally approved by the FDA in 2016 but not by the European Medicines Agency (EMA); therefore, it is not marketed in Europe. Of note, the manufacturer is obliged to present the data from the next randomized trial before May 2021 and those results will determine whether the initial doubts were unfounded [79].

As indicated in Table 1, a leading company, Sarepta Therapeutics, is actively working in the field of splice modulating ASOs. Recently, after positive results of phase 1–2 trials and the ongoing phase 3 trial, the FDA approved another drug, called golodirsen (VYONDYS 53, SRP-4053). This ASO is predicted to be used in around 8% of DMD patients having a confirmed exon 53 amenable mutation. Casimersen (previously named SRP-4045), the morpholino antisense, after promising results from phases 2 and 3 trials (ESSENCE trial, NCT02500381), showing a significant mean increase in dystrophin protein in the casimersen-treated group, is going to be tested further in DMD individuals amenable to exon 45 skipping in the phase 3 extension study (NCT03532542) by weekly intravenous infusions at 30 mg/kg for up to 144 weeks (golodirsen-treated patients are also included in this trial). As an example of studies performed by other companies, the safety, tolerability, and clinical efficacy of NS-065/NCNP-01 (viltolarsen) in patients amenable to exon 53 skipping is performed by NS Pharma. In contrast to other morpholino oligomers given intravenously, DS-5141b, an antisense oligonucleotide consisting of 2′-*O*,4′-C-ethylene-bridged nucleic acids and 2′-*O*-methyl RNA, targeting exon 45 is going to be injected subcutaneously (Table 1). On 25 March 2020, the drug received its first approval in Japan [80], whereas, on 7 February 2020, the company announced that the FDA accepted its New Drug Application (NDA) seeking approval under Priority Review and the decision should be released in the third quarter (July–September) of 2020. If accepted, it will be the next drug, after golodirsen, for the treatment of DMD in patients amenable to exon 53 skipping in the USA, and globally.

The systemic therapeutic effects of this technology may be limited by the endosomal entrapment observed both in muscle and cardiac tissues. Moreover, Alter et al. have shown that its effectiveness in the heart is lower than in skeletal muscles, as even after a high-dose multi-injection delivery, the restoration of dystrophin expression was observed in different muscles, but not the cardiac tissue [81]. A lack of restoration of dystrophin expression in the heart, along with improved skeletal muscle function, may, paradoxically, exacerbate cardiomyopathy due to increased physical activity of DMD patients (this might be a general problem of therapies, which concentrate only on skeletal muscles without targeting cardiac tissue) [82]. Therefore, improved cardiac targeting/delivery of ASOs to the heart is a challenge for the future. Several strategies have been described recently, including chemical modifications and new formulations, like tricyclo-DNAs, nanoparticles, peptides, and polymers (reviewed in [20]). For example, conjugation of PMOs to peptides (PPMOs) enhances cell permeability and increases the expression of dystrophin in the hearts of dystrophic mice [83, 84].

Continuous development and modifications in ASO-based therapy led to the suggestion of using cocktail ASOs or the multiple exon skipping (or multi-exon skipping) to restore the dystrophin mRNA open reading frame. Such a strategy might be potentially applicable to 80–90% of DMD patients in total, regardless of mutation type [85, 86]. This interesting therapeutic approach for treating DMD was first tested in the canine X-linked muscular dystrophy (CXMD) dog model, harboring a splice site mutation in intron 6, leading to a lack of exon 7 in dystrophin mRNA. The approach, based on the multi-exon skipping of exons 6 and 8, led to the correction of the reading frame and resulted in the truncated dystrophin expression in skeletal muscles [87]. Further studies showed that PPMOs cocktail designed to skip dystrophin exons 6 and 8 after four systemic administrations into CXMD dogs rescued dystrophin expression in the myocardium and cardiac Purkinje fibers and improved cardiac conduction abnormalities in the dystrophic heart [88]. Those results indicate the effective applicability of ASOs in both muscle and cardiac DMD dysfunctions, hopefully as a routine treatment of DMD patients in the near future.

### Readthrough therapy

So-called readthrough therapy relies on restoring dystrophin expression through the inhibition of translation termination of a nonsense mutation. One of the first compounds with such properties, tested in vitro [89] and in vivo, in *mdx* mice [90, 91], was gentamicin, the antibiotic able to read through a nonsense mutation. Those preclinical studies gave divergent outcomes; however, several clinical trials for DMD with the use of aminoglycoside therapy were initiated. The results from the phase 1 study conducted on 16 subjects with documented stop codon mutations receiving weekly (*n* = 12) or twice weekly (*n* = 4) gentamicin for 6 months indicated an increase in the dystrophin expression is some patients, with the most significant level detected in three patients (13.0–15.4% of wild-type levels, the effect irrespective of the regimen and with independent concordance by immunofluorescent and western blot analyses). Of note, a statistically significant decrease in serum CK level after 6 months of gentamicin treatment was detected. However, a crucial indicator of the improvement in the quality of life like muscle functioning was not changed [92].

Long-term administration of gentamicin is related to its ototoxicity and nephrotoxicity [93]. Therefore, gentamicin derivatives NB74 and NB84 with superior activities in terms of cell toxicity and readthrough efficiency over gentamicin have been tested in vitro [94]. Also another aminoglycoside antibiotic NPC-14 (arbekacin sulfate), an inhibitor of 30S ribosomal subunit, resulting in codon misreading and inhibition of translation was evaluated in the so-called NORTH POLE DMD trial at Kobe University (NCT01918384).

The most promising drug restoring the expression of functional dystrophin by reading through the premature nonsense stop signals on dystrophin mRNA is ataluren (3-[5-(2-fluorophenyl)-[1,2,4]oxa-diazol-3-yl]-benzoic acid). It can be potentially used in individuals with a nonsense mutation in the *DMD* gene (around 11% of boys). Bushby et al. described the result of a phase 2b study (NCT00592553) in which patients received ataluren orally three times daily for 48 weeks (10, 10, and 20 mg/ kg referred to as ataluren 40 mg/kg/ day; or 20, 20, and 40 mg/kg, referred to as ataluren 80 mg/kg/day) or a placebo [95], (Table 1**)**. Ataluren 40 mg/kg/day treated patients performed better than placebo patients, in contrast to individuals receiving the higher dose. It indicates a bell-shaped dose–response relationship which was already suggested by phase 2 study (NCT00264888) [96]. Results from the phase 3 trial (NCT02819557) performed in boys aged 7–16 receiving ataluren orally three times daily (40 mg/kg/day) for 48 weeks in comparison to placebo-treated patients did not show significant changes in 6MWD. However, some effect of ataluren was noted in the prespecified subgroup of patients with a baseline 6MWD of 300 m or more to less than 400 m [97]. Based on those and other studies (there are 16 clinical trials registered for ataluren and DMD), the drug has received conditional approval in the European Union [14, 98], but the same data were not sufficient to register it by the FDA. Currently, a long-term observational study of ataluren (Translarna) safety and effectiveness in usual care is ongoing. The results from this post-approval safety study, intended to enroll approximately 200 patients across ~ 60 care centres in Europe who will be followed for at least 5 years from their date of enrolment, should bring more information about the drug safety and effectiveness in routine clinical practice.

### CRISPR/Cas9 gene editing as a promising tool for DMD treatment

**Fig. 6:**
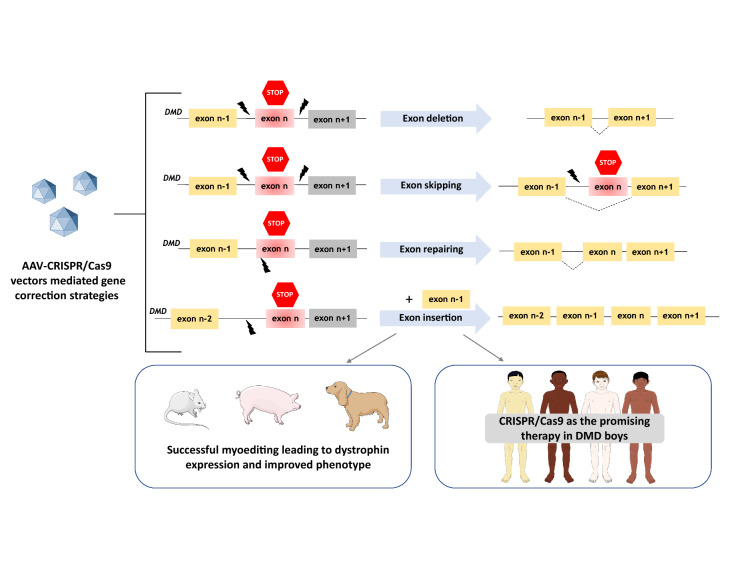
CRISPR/Cas9 technology for myoediting. AAV-CRISPR/Cas9 vectors have been used in mice, pigs, and dogs to correct *DMD* mutations through different strategies, including exon deletion, exon skipping, exon repairing, or exon insertion. In case of CRISPR/Cas9-based exon skipping, the indel introduced by Cas9 allows skipping of the mutated exon during mRNA maturation. For more details on possible strategies and their mechanisms, consult the paper by Min et al. [100]

In recent years, the CRISPR/Cas9 system has been adapted as a tool that can edit the genome of nearly any organism and repair various genetic defects, including also the correction of mutated *DMD* gene. Functional dystrophin gene restoration has been demonstrated by CRISPR/Cas9 editing in myoblasts differentiated from induced pluripotent stem cells (iPSCs) of DMD patients [99, 100] [described in more detail in “Generation of myoblasts from embryonic stem cells (ESCs) and induced pluripotent stem cells (iPSCs)”]. By delivery of CRISPR/Cas9 components (Cas9 mRNA, a sgRNA targeting the mutated exon 23, and an ssODN repair template) into the zygotes of *mdx* mice, it was demonstrated that CRISPR/Cas9-mediated editing (termed myoediting [101]) can successfully correct *Dmd* mutation by homology-directed repair—HDR or by nonhomologous end-joining—NHEJ and restore dystrophin expression [102]. In 2016, three separate groups published results demonstrating the usefulness of this method for the restoration of dystrophin expression in adult mouse models of DMD [103–105]. A similar study was performed later in dystrophic dogs [106] and recently, Moretti et al. [107] applied CRISPR/Cas9 gene editing in dystrophic pigs. In the above studies, CRISPR/Cas9 components were delivered in vivo using AAV vectors with quite positive results [103–108], (Fig. 6). For example, after i.p. injections of the AAV vector into neonatal mice, recovered dystrophin expression was present not only in abdominal muscles but also in the diaphragm and heart. When intravenous administration was performed in 6-week-old adult *mdx* mice, prominent recovery of dystrophin was found in the cardiac muscle [103].

These studies indicate the therapeutic potential of the CRISPR/Cas9 system in the DMD therapy and emphasize that a single systemic treatment can be directed to a large number of cardiomyocytes and, thus, has a protective effect on the heart. However, to increase the targeting efficiency of the CRISPR/Cas9 system, the usage of a higher dosage of AAV was proposed. El Refaey et al. [108] compared low (3 × 10^11^ vg/mouse) *vs* high dose (1 × 10^12^ vg/mouse) of systemic delivery of AAV-SaCas9/gRNAs in 3-day-old *mdx/Utr*^+/−^ neonates. After 10 weeks of treatment, almost no dystrophin-positive fibers were detected in the low -dose group, whereas about 40% of cardiomyocytes were dystrophin positive in the high-dose treatment group. Moreover, the rate of heart fibrosis was potently decreased, whereas cardiac contractility was improved after in vivo genome editing [108]. Similarly, in the porcine *DMD*Δ52 model, a low dose (1–2 × 10^13^ virus particles per kg (vp/kg) coated with G2-AAV9-Cas9-gE51, sporadically transduced skeletal muscle specimens, whereas a high dose (2 × 10^14^ vp/kg) led to the prominent dystrophin protein expression in skeletal muscles, the diaphragm, and heart [106].

The concern on CRISPR/Cas9 editing, especially for therapeutic and clinical applications, is related to the high frequency of off-target activity [109, 110], including the risk for mutations at sites other than the intended. The repair of double-strand break-induced by CRISPR/Cas9 could also lead to large deletions and/or complex rearrangements [106, 107]. Taking into account the possibility of pathogenic consequences of CRISPR/Cas9 editing, recent studies have been aimed at evaluating the long-term effectiveness and safety of this therapy for DMD treatment. Xu et al. [108] have checked the outcome of myoediting (deletion of the exons 21–23 to restore the dystrophin reading frame) after 19 months of delivery of AAVrh74-SaCas9/gRNA into 3-day-old *mdx* pups. No serious adverse effects of CRISPR genome editing (including no signs of tumorigenesis) were observed when various organs were analysed. Moreover, the large deletion events were not identified in the AAV-CRISPR-treated heart samples. Of note, the beneficial effects like restored dystrophin expression and improved cardiac function were evidenced. Similar long-term experiments, assessing the effectiveness and safety of CRISPR/Cas editing, were performed by Nelson et al. [109] and Hakim et al. [110]. Systemic administration of a single dose of AAV-Cas9 in neonatal mice led to dystrophin protein expression 1 year after treatment [109], whereas an optimization (increase) of the gRNA vector dose resulted in a higher level of the total dystrophin transcript in comparison to untreated *mdx* mice and the improvement in cardiac electrophysiology and hemodynamic parameters even 18 months after a single intravenous of CRISPR/Cas9 vector [110]. In the future, similar experiments evaluating the long-term potency and safety issues should be performed on non-mouse models.

### RNA interference

Although not frequently used, strategies utilizing the RNA interference approach have been described in the literature and even tested in clinical trials. The combined therapy, utilizing dystrophin upregulation through an exon skipping approach and RNA interference against the activin receptor type IIb (ActRIIB) [111]—the receptor for myostatin—or the soluble version of this receptor was tested in dystrophic animals [112, 113]. Myostatin, known also as growth differentiation factor 8 (GDF-8), belonging to the superfamily of TGF-β signaling molecules, acting via specific transmembrane receptors, mostly ActRIIB was shown to play a role in DMD through regulation of muscle cell growth and differentiation [113]. Myostatin or ActRIIB inhibition via different tools (not only RNA interference but also exon skipping, anti-myostatin/ActRIIB antibodies, dominant-negative myostatin/ActRIIB, pharmacological inhibition, etc.) has revealed beneficial effects on muscle mass and function in wild-type and dystrophic *mdx* mice (reviewed in [113]). In contrast, several reports did not find any improvement after blocking myostatin/ActRIIB signaling in humans undermining the effectiveness of this therapy [114–116]. Of note, two clinical trials with ACE-031, a soluble form of the human ActRIIB, were terminated based on the preliminary safety data (Table 1). Similarly, the trials conducted with myostatin antibodies (performed by Roche—NCT03039686 and Pfizer—NCT02907619, Table 1) also failed to meet the primary endpoint, arguing the usefulness of this strategy in DMD patients. However, in a recent study, Mariot et al. [117] have suggested that the limited effectiveness of anti-myostatin approaches is related to low myostatin levels detected in DMD patients. A decreased mRNA level of the myostatin pathway in muscle biopsies, as well as low levels of circulating myostatin in several neuromuscular diseases, were found. Moreover, when the level of myostatin was compared in patients with and without cardiac symptoms, it turned out that DMD individuals with cardiomyopathy had significantly lower myostatin levels than patients without heart problems [118]. Therefore, it was suggested that the inhibition of this pathway by an exogenous compound (monoclonal antibody or vector-mediated inhibition) does not lead to an improvement in phenotype and strongly limits the potential clinical efficacy of this approach. Interestingly, the myostatin level in *mdx* mice is at least 50 times higher than in human DMD individuals [118]. This could be one of the reasons for the differences in the reported effectiveness of anti-myostatin therapy in a mouse model of the disease [112, 113, 119, 120] and humans [114–116].

### Dystrophin-independent gene therapies

In addition to dystrophin-based therapies, other muscle-stabilizing proteins, such as follistatin, GALGT2, and biglycan, have been tested as the experimental genetic treatments. Moreover, calcium regulation using AAV-SERCA and AAV-nNOS gene transfer approaches has been conducted.

Several studies have shown the beneficial effects of myostatin inhibition through the utilization of gene therapy with follistatin (FS), a factor regulating muscle regeneration [118, 119]. A one-time gene administration (1 × 10^11^ AAV1-FS) to 3–4-week-old *mdx* animals resulted in long-term effectiveness, found even after 2 years. Of importance, when similar treatment was performed in older mice (6.5 months old), enhanced muscle strength was still observed. Before starting clinical trials, a similar strategy was tested in nonhuman primates. Kota et al. have shown the safety of intramuscular injection of AAV1 vector expressing the human FS344 transgene, which encodes the FS315 follistatin isoform (AAV1-FS344) into cynomolgus macaque monkeys [118]. In another study, concomitant delivery of microdystrophin and follistatin [120] was much more effective in improving muscle force than sole therapy, even in old animals (600-day-old *mdx* mice). Those results support the usefulness of combination therapy with gene replacement and muscle enhancement in DMD treatment. In 2015, results from a phase 1/2a follistatin gene therapy trial were published. In two cohorts of BMD patients, after intramuscular injection of 3 × 10^11^ vg/kg/leg or 6 × 10^11^ vg/kg/leg, an improvement in 6MWD was observed in four out of six analysed individuals and overall reduced endomysial fibrosis and centrally nucleated fibers indicated positive effects of the therapy [121]. However, to the best of our knowledge, the effects of such treatment on mitigating the DMD cardiac phenotype have not been investigated yet.

AAV virus serotype rh74 carrying the GALGT2 gene under the control of an MCK promoter (rAAVrh74.MCK.GALGT2) was developed by Sarepta Therapeutics. The GALGT2 gene encodes glucosyltransferase capable of upregulating the expression of various surrogate genes and proteins, including utrophin, agrin, laminin α4, laminin α5, integrin α7, and integrin β1. Moreover, it encodes β1, 4 N-acetylgalactosaminyl transferase that glycosylates α-dystroglycan in skeletal muscle. Its overexpression was shown to have beneficial effects in mouse models of various dystrophies: in *mdx* mice [121], the dyW model for congenital muscular dystrophy [122], and the Sgca^−/−^ model for limb-girdle muscular dystrophy 2D (LGMD2D) [123]. Not only was skeletal muscle damage ameliorated by this therapy, but it was found to have improved heart function and cardiac output in mice overexpressing GALGT2 in comparison to mock-treated animals. In treated *mdx* mice increased expression of utrophin protein and a higher glycosylation level of α-dystroglycan protein was determined in the cardiac tissue [124]. These preclinical studies led to the conduction of a phase 1/2 clinical trial (NCT03333590) evaluating the safety and effectiveness of rAAVrh74.MCK.GALGT2 delivery to lower limbs through the femoral artery using an intravascular limb infusion technique (ILI). The results are expected in November of 2021, after regular evaluation of the patients for up to 24 months. Further experiments have to be performed, but this strategy may be potentially successful in DMD of various origin (regardless of the mutation), as although it does not restore dystrophin, it recruits dystrophin surrogates to compensate for the lack of dystrophin and protect the cell membrane.

Biglycan is a small leucine-rich extracellular matrix protein that interacts with multiple components of the dystrophin complex including dystroglycan and sarcoglycan components. Its non-glycanated form (NG-biglycan), able to induce the localization of nNOS and utrophin to the muscle membrane [125, 126], was evaluated in the dystrophic animal model. In *mdx* mice, the use of both recombinant biglycan [126], as well as biglycan in the form of gene therapy [118], resulted in improved structure and function of skeletal muscles with no sign of toxicity. Recombinant AAV8 carrying hBGN encoding human biglycan was intravenously injected into 5-week-old *mdx* mice. Not only did it decrease the disease biomarker, CK level, but the number of central myonuclei and the distribution of myofiber sizes, as well as motor functions, also improved. Following encouraging preclinical results, Tivorsan Pharmaceuticals manufactured a highly purified formulation of this ECM protein and was planning to initiate clinical trials with TVN-102 in early 2017/2018; however, so far, no results are available.

As mentioned in “Duchenne Muscular Dystrophy: an overview”, neuronal nitric oxide synthase (nNOS) represents an important part of the DGC complex and dystrophin deficiency results in disturbed nNOS-NO signaling [5–7]. Accordingly, nNOS gene therapy was tested as a possible DMD treatment. Lai et al. [127] constructed AAV9 vectors containing shortened nNOS coding sequence, namely, lacking a PDZ domain in the nNOS gene (ΔPDZ nNOS). Delivery of such vector to the heart of aged (∼14-month-old) *mdx* mice led to the improved heart function with reduced myocardial fibrosis, inflammation, and apoptosis evident in mice at 21 months of age. Not only NO signaling but also the homeostasis of Ca^2+^ is imbalanced in dystrophic muscles. The increased cytosolic calcium may lead to myofiber death and muscle dysfunction and it may greatly affect the functioning of cardiomyocytes. Therefore, the normalization of the calcium signaling in DMD as the treatment strategy has been proposed. One of the attempts is to overexpress sarco/endoplasmic reticulum (SR) calcium ATPase (SERCA), a calcium pump that transports cytosolic calcium to the SR during excitation–contraction coupling. The benefits of such treatment have been demonstrated in various animal models of DMD. The disease was mitigated in both dystrophin mutant *mdx* mice and δ-sarcoglycan–null (Sgcd^–/–^) mice after overexpression of both SERCA1a and SERCA2a isoforms (both isoforms are found in adult muscle; however, SERCA1 is selectively expressed in skeletal muscle, while SERCA2a is present either in skeletal or cardiac muscle) [128]. In this study, 3-day-old *Sgcd*^*–/–*^ pups were injected either with 10^10^ viral particles of the therapeutic gene (AAV9-SERCA2a) or with the control vector (AAV9-GFP), and analysis performed 6 weeks later demonstrated attenuation of dystrophic phenotype. Other studies have reported the beneficial effects of neonatal AAV2/6-SERCA1a overexpression in the diaphragm [129] as well as cardiac improvements after AAV9-SERCA2a delivery to 12-month-old *mdx* mice (1 × 10^12 ^vg particles/mice) [130]. Long-lasting effects of such therapy were studied by Wasala et al. [131], and the amelioration of cardiomyopathy and skeletal muscle protection was demonstrated after 18 months after a single systemic delivery of SERCA2a isoform (AAV9-SERCA2a; 6 × 10^12^ vg particles/3-month-old mdx mouse). SERCA2 was highly overexpressed in the heart and skeletal muscle causing an increase in SR calcium uptake, better physical performance, no signs of myocardial fibrosis, and the improvement in ECG and restoration of ejection fraction in treated dystrophic animals.

## Cell therapies

The idea of cell-based therapies relies on the transplantation of cells expressing functional dystrophin obtained from an unaffected donor (allogeneic transfer) or a patient after ex vivo genetic modification. The ideal cells should be delivered systematically to affect not only limb muscles but also the heart and diaphragm; should cross the blood vessel wall and be able to easily reach the muscles from the bloodstream (to avoid multiple intramuscular injections); should integrate into resident myocytes and self-renew to provide a long-term effect without inducing an immune response. Several various cell populations have been studied until now (Fig. 3); however, the usage of some cells, including bone marrow-derived mesenchymal stem cells (BM-MSCs) and CD133 + progenitors [132–134], is questionable. Although their limited ability to differentiate into muscle cells was described [135], follow-up experiments showed that only a small number of the BM-MSCs were capable of contributing to muscle fiber formation (in comparison to controls where muscle‐derived myoblasts were implanted) [136]. This observation suggested that the therapies with satellite cells (SCs) and other muscle-derived stem cells (MDSCs) are superior as the cells are more suited to participating in new fiber formation. Moreover, mesoangioblasts, pericyte-derived cells, and myogenic progenitors differentiated from embryonic stem cells (ESCs) and induced pluripotent stem cells (iPSCs) have been tested. Unfortunately, the promising results from in vivo studies with some of these cells were not recapitulated in human trials [137, 138], while the others, based on pluripotent stem cells, still await testing in clinical trials.

### Myoblast and satellite cell transplantation

Satellite cells (SCs), mononuclear cells with low cytoplasmic content, expressing Pax7 transcription factor, are located between the basal lamina and sarcolemma of adult skeletal muscle fibers [139, 140]. At least a part of these cells is able to self-renew and the cells are considered bona fide muscle stem cells, able to form new muscle tissue. After activation (mostly in response to injury), quiescent SCs proliferate and differentiate into myoblasts, which fuse with each other or with existing myofibers to repair the damaged muscles. This ability suggests that SCs are perfect for the treatment of muscle loss or disease, including DMD.

One of the first studies performed in the late 1980s gave very promising results and demonstrated the feasibility of normal myoblast injections into the muscles of *mdx* mice to correct a biochemical defect [141]. Some other studies also detected the expression of dystrophin-positive fibers in dystrophic individuals after normal myoblast delivery [142, 143]. Unfortunately, clinical trials did not bring optimistic results [144, 145]. Miller et al. [145] injected 100 million allogeneic myoblasts in the anterior tibial muscle of one leg and a placebo in the other leg of 10 DMD boys. Increased force generation was found in both legs, but dystrophin expression was detected only in three patients after 1 month and in one patient after 6 months. As cyclosporine was administered for 7 months after myoblast injection, these results indicate that improvement in muscle function was rather due to immunosuppressive drug treatment and was not related to the cell-mediated effect. The results of a study performed by Mendell et al. [144] were even less promising, although the general regimen was very similar to the previous study (110 million cells injected once a month for 6 months to the biceps brachii muscles of one arm of each of 12 DMD boys with the other arm serving as sham-injected controls receiving cyclosporine or a placebo). No improvement in the muscle strength in the arm injected with myoblasts and dystrophin expression analysed by imunostaining found in one patient at ~ 10% and less than 1% in three other patients cannot be considered as a positive outcome of the therapy. As the study by Miller et al. [145] suggested a beneficial effect of immunosuppression, the next attempts were performed to combine such treatment with an increased frequency of cell injections and/or higher cell numbers. Several papers by Skuk et al. [146–148] have described the new protocol based on the so-called “high-density injection” (the number of injections varied between 25 and 200) together with tacrolimus immunosuppression, which resulted in even 30% of the donor-derived dystrophin-expressing myofibers visualized by fluorescent immunodetection.

Unfortunately, the poor survival and limited migration of myoblasts in vivo indicate that they are not the best option for cell-based therapies. SCs could be good candidates; however, quick differentiation of human cells to myoblasts limits their regenerative potential [149].

### Mesoangioblasts

Mesoangioblasts, vessel-associated progenitors, able to differentiate into muscle fibers [150] were tested in mouse [151] and canine model [152] of DMD. Although critically evaluated by Bretag [138], the original study showed that intra-arterial delivery of dystrophin-expressing mesoangioblasts resulted not only in the recovery of dystrophin expression but also in the improved muscle morphology and function of dystrophic dogs [152]. This and other studies pointed out the important ability of mesoangioblasts to cross the blood vessel wall. Such an advantage over SCs and myoblasts, which need to be delivered directly into the muscle tissue to properly engraft, indicates that these cells are valuable therapeutic cells [153, 154].

An interesting approach was described by Tedesco et al. [155], who used a combination of a human artificial chromosome (HAC)-mediated gene replacement and transplantation with blood vessel-associated stem cells. Mesoangioblasts from dystrophic mice after genetic correction with a HAC vector containing the entire (2.4 Mb) human dystrophin gene were injected into *mdx* mice. The morphological and functional amelioration of the dystrophic phenotype that lasted for up to 8 months after transplantation indicated the effectiveness of HAC-mediated gene transfer. Another example of mesoangioblast modification utilizes PiggyBac transposons. This system was more efficient in terms of stable gene transfer to primary mesoangioblasts when compared to the transfection of plasmid vectors and it resulted in satisfactory transgene expression when transplanted intramuscularly into dystrophic animals [156, 157].

Those and other studies suggested that mesoangioblasts could be a valuable approach for stem cell therapy for Duchenne patients. However, a phase 1/2a clinical trial performed on five DMD patients revealed dystrophin expression only in one individual. Moreover, magnetic resonance imaging documented the progression of the disease in four out of five patients. Additionally, this trial indicated problems with the safety of mesoangioblast infusion as one patient developed a thalamic stroke [158], limiting the potential use of this approach in clinics.

### Generation of myoblasts from embryonic stem cells (ESCs) and induced pluripotent stem cells (iPSCs)

As indicated above, cell therapies still have not given the expected effects. Therefore, new possibilities based on pluripotent stem cells, such as embryonic stem cells (ESCs), may be taken into consideration. ESCs, derived from the inner cell mass of the pre-implantation blastocyst stage, can be differentiated into all three germ layers of the embryo [159]. Different protocols can be applied to obtain SCs from ESCs and one of them involves the conditional overexpression of the Pax7 transcription factor [160]. By the use of a doxycycline-inducible lentiviral vector encoding Pax7 and after intramuscular transplantation of such modified progenitors to immunodeficient *mdx* mice, widespread engraftment, shown by a large number of myofibers expressing human dystrophin, was evident [160].

However, the ESCs’ approach raises ethical controversies, and because of law regulations, it is not allowed in many countries. Therefore, obtaining the desired cell type from the induced pluripotent stem cells (iPSCs) creates a new possibility also for DMD modelling. Described for the first time in 2007 by Shinya Yamanaka, the 2012 Nobel Prize winner, human iPSCs (hiPSC) are generated from easily accessible somatic cells by overexpression of defined transcription factors (most often OCT4, SOX2, KLF4, and c-MYC) [161]. In therapeutic perspectives, patient-derived iPSCs can be genetically corrected, differentiated in vitro into, for example, muscle cells and such dystrophin-expressing cells administered to the patient without inducing an immune response.

Although very attractive, a limitation of this method is the way of delivery of iPSC-derived muscle cells. Till now, intramuscular administration is the most efficient way [162], but systemic delivery, if shown feasible, would possess several benefits, such as the reduction of the number of injections and the possibility to target not only selected limb muscles but also the heart and diaphragm.

iPSCs’ technology may be of particular interest when heart problems are evaluated. As mentioned earlier, dystrophin-deficient cardiomyocytes exert serious functional problems and dilated cardiomyopathy greatly contributes to the mortality of DMD patients [19]. Therefore, the understanding of cardiomyocytes functioning, as well as the correction of defective cardiomyocytes obtained from DMD patients, should be of great importance. However, it is difficult to model this pathology in vitro as cardiac tissue is highly inaccessible [163] and the collection of a heart biopsy is not a routine diagnostic procedure [164]. iPSCs differentiated to cardiomyocytes may help to solve these problems as it has been already demonstrated that patient-derived iPSCs-cardiomyocytes (iPSCs-CMs) recapitulate the pathophysiological phenotype observed in several cardiac diseases and may serve as a tool for investigating the molecular basis of a particular heart condition as well as for drug screening (reviewed in: [165]).

Cells of DMD patients have been reprogrammed to iPSCs, differentiated to the cardiomyocytes and skeletal muscle, and were shown to recapitulate the DMD phenotype [99, 166–168]. More important, even the very large *DMD* gene deletions in such cells can be corrected by the CRISPR/Cas9 editing strategy, generating isogenic cells [99, 168]. Such strategy was utilized by Lin et al. who used human DMD iPSCs corrected by TALENs and CRISPR/Cas9 technology (through exon 45 skipping, frameshifting, and exon 44 knocking) and differentiated to skeletal muscle cells. The expression of full-length dystrophin protein in corrected cells was evident [169]. However, a combination of gene editing with autologous iPSC-derived myoblasts remains to be tested in clinical trials.

**Fig. 7:**
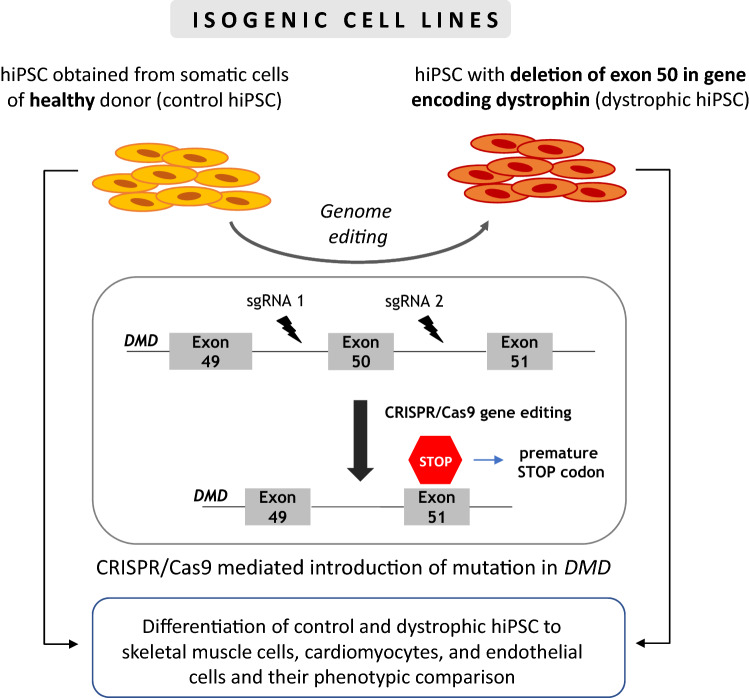
Generation of the isogenic iPSCs cell line with CRISPR/Cas9 technology for studying the mechanism of DMD. The design of sgRNA targeting sequences located upstream and downstream of exon 50 leads to its deletion and creation of the isogenic cell line differing only at this locus from the control human iPSCs line. Further differentiation to skeletal muscle cells, cardiomyocytes, and endothelial cells allows the phenotypic comparison between normal and dystrophic cells

The possibility of the generation of the isogenic cell line, differing only at the locus of interest, eliminates potential problems when comparing cells from various patients and healthy donors, which may be related to genetic background, sex, and ethnicity of patients, as well as other characteristics. In the context of studying mechanisms of DMD progression, modifying the iPSCs from healthy donors to introduce mutation is a valuable tool. The method used in our study is based on the design of specific sgRNAs targeting sequences located upstream and downstream of exon 50 (as deletion of exon 50 is one of the most common mutations in DMD patients). This allows removing exon 50 of *DMD* in control iPSCs line, further differentiation to desired cell type, and the downstream analysis of control and dystrophic cells (Fig. 7).

Finally, in the future, iPSC-CMs can be applied for the treatment of DMD cardiomyopathy. Replacing damaged cardiomyocytes by CRISPR/Cas9 corrected autologous iPSC-CMs or allogeneic iPSC-CMs are the potential option in light of recent studies showing the improvement of heart function after pluripotent stem cells derived CMs transplantation in animal models of heart failure ([170, 171], Stepniewski et al., submitted). However, this approach is medically and technically challenging, and will require to solve the problem of necessary immunosuppression (due to dystrophin and allogeneic cardiomyocyte immunogenicity) and risk of arrhythmia.

### Other cells used in cell therapy for DMD

Various cell-mediated therapies for DMD have been tested in recent years; however, clinical trials with stem cells are limited and up until now use mostly bone marrow and human umbilical cord cells, whose usage for treatment of DMD may be highly disputable. Other candidates, but still under evaluation, are cardiosphere-derived cells (CDCs), claimed to be a cardiac progenitor cell population, tested in *mdx* mice and even in clinical trials of acquired and congenital forms of cardiomyopathy and in patients with heart failure and reduced ejection fraction [172, 173]. In *mdx* mice, intramyocardial delivery of CDCs improved not only the heart but also skeletal muscle structure and functions including better exercise capacity and increased survival [174]. The authors suggest the modulation of Akt, Nrf2 and NF-κB signaling pathways by CDCs and CDCs exosome. Also, the so-called Halt Cardiomyopathy Progression (HOPE)-Duchenne trial was performed in 13 DMD individuals (mean age 18.7 years) receiving systemic corticosteroid therapy and other cardioprotective drugs as well as allogeneic CDCs (CAP-1002) (Table 1, NCT02485938, [175]). The initial results showed improvement in regional cardiac and skeletal functions after one intracoronary delivery of CAP-1002, but further studies should give more information about this kind of therapy in DMD patients. However, in the light of controversies and recent stringent studies falsifying the existence of cardiac stem cells [176–178], the scientific rationale for the described approach should be critically evaluated.

Other options (not requiring the stem/pluripotent stem cells) being tested include the generation of myoblasts directly from patients’ fibroblasts. The conversion of somatic cells (fibroblasts) into myogenic cells may be forced by the overexpression of the myogenic factor MyoD, and some studies showed fusing of such myoblasts with existing muscle fibers in mice [179–181]. Ito et al. [182] have found that defined combinations of transcription factors (Pax3*,* Mef2b, and Pitx1 or Pax7, Mef2b, and Pitx1 in embryonic fibroblasts, and Pax7*,* Mef2b, and MyoD in adult fibroblasts) efficiently reprogrammed murine fibroblasts into skeletal muscle progenitor cells able to form dystrophin-positive mature muscle fibers when transplanted into *mdx* mice. Lee et al. [183] described the method of murine fibroblasts conversion to induced myogenic stem cells (iMSCs), characterized by the effective differentiation into multinucleated myotubes and higher proliferation capacity than muscle-derived stem cells. It was demonstrated that the combination of four transcription factors, Six1, Eya1, Esrrb, and Pax3, is critical to establish iMSCs [183]. Another strategy was proposed by Bar-Nur et al. [184]. iMSCs, generated by the combination of the transient MyoD transcription factor expression and small-molecule treatment (the cyclic AMP agonist forskolin, the TGF-β inhibitor RepSox and the GSK3β inhibitor CHIR99021), were able to self-renew and expressed markers of muscle stem, progenitor, and mature cells. Finally, the cells were able to differentiate into dystrophin-positive myofibers in *mdx* mice and contribute to muscle regeneration in a serial injury model (barium chloride-induced injury in immunodeficient Foxn1^nu^ mice). More clinical studies with iMSCs are needed to fully check their application as a muscle regenerative therapy for muscle-wasting DMD disease.

## Pharmacological therapies

Many pharmacological compounds are under evaluation both in the *mdx* mice as well as in clinical trials, and they are used to fight against various processes contributing to the disease’s pathology. Not only are anti-inflammatory factors or utrophin upregulators examined, but compounds/drugs regulating calcium signaling dysregulation, increased oxidative stress, mitochondrial dysfunction, accumulation of fibrosis, and defective angiogenesis are also under constant screening (Fig. 3). As several reviews describing in detail the pharmacological strategies used in DMD have been published recently [185, 186], we will only present a summary of the achievements in this field (Table 1) and concentrate on the selected approaches.

### Inhibition of NF-κB signaling exerts positive effects in ameliorating dystrophic pathology

Glucocorticoids (GCs) acting as anti-inflammatory agents are frequently used to halt progressive muscle damage; however, they concomitantly exert many adverse events leading to, among others, weight gain, loss of bone density and osteoporosis, diabetes, and muscle atrophy [187]. Prednisone, prednisolone, and deflazacort, mostly through inhibition of NF-κB signaling, have been shown to exert long-term protective effects and serve as a gold standard for the treatment of DMD [188, 189]. To decrease possible side effects, Quattrocelli et al. [190] have suggested weekly (instead of daily) steroid treatment and found comparable protective effects of both regimens, but only less frequent administration did not elicit muscle atrophy. GCs have been tested in many clinical trials (Table 1) and recent studies suggest that these drugs may improve not only muscular but also cardiac functions in dystrophinopathies [191–193].

GCs act mostly as anti-inflammatory drugs. It has to be underlined that further studies, with new drugs, lacking unwanted secondary effects are still warranted. Vamorolone (previously known as VBP15) may be a good example of such a compound. This glucocorticoid analog investigated by ReveraGen has been shown to exert superior activities in comparison to standard GCs. Heier et al. [194] found that vamorolone-treated *mdx* mice exert increased grip strength and decreased inflammation whereas such treatment did not exert side effects observed with prednisolone (e.g., stunted growth of developing mice, and increased muscle fibrosis in skeletal or cardiac muscle). A phase 1 trial performed in healthy adults gave promising results as any pharmacodynamic safety concerns, typical for GCs, were noted up to 20 mg/kg/day [195]. Recent results from the study performed on 48 boys with DMD (aged 4 to  < 7 years, steroid-naive), treated daily with different doses of vamorolone, showed efficacy and lack of serious side effects at doses of 2.0 and 6.0 mg/kg/day in a 24-week-treatment period [196, 197] (Table 1). Of importance, vamorolone, being a mineralocorticoid receptor antagonist and glucocorticoid receptor agonist, may also exert cardioprotective effects [198].

### Pharmacological interventions targeting histone deacetylases

The degeneration of dystrophic muscles suggests that compounds able to increase the regenerative potential of skeletal muscle and preventing the replacement with inflammatory infiltrate, and fibrotic and fat tissue may ameliorate disease progression. Interestingly, epigenetic modifiers might exert such beneficial effects through enhancement of the regenerative potential of dystrophic muscles while preventing their degeneration. One of the examples of such regulators are histone deacetylases (HDAC) which catalyze the removal of acetyl groups from lysines within histone and nonhistone proteins. HDAC activity was shown to be upregulated in dystrophic muscles and the effectiveness of HDAC inhibitors (HDACi) was demonstrated in *mdx* mice [199–201]. The mechanism of HDACi action may include an increase in the level of follistatin, a factor regulating muscle regeneration [202]. As mentioned in “Dystrophin-independent gene therapies”, follistatin is known to decrease the expression of myostatin, an inhibitor of muscle fibers regeneration but may also act through pathways independent of the myostatin signaling cascade [203]. Initial studies evaluated the structurally unrelated HDAC (drugs which were already used in clinical practice for different therapeutic indications)—trichostatin A (TSA), valproic acid (VPA), and phenylbutyrate (PhB)—and indicated that mostly TSA restores muscle function and morphology in dystrophic animals [199]. Another example of HDACi tested in *mdx* mice is suberoylanilide hydroxamic acid (SAHA). This compound was effective in the amelioration of disease progression at doses between 0.6 and 5 mg kg/day delivered to *mdx* animals for 3 months [204]. Finally, the number of studies performed with another compound blocking the activity of HDAC, givinostat, led to phase 3 clinical trials (NCT03373968, NCT02851797). Givinostat was first evaluated in dystrophic mice, and in this model, its effectiveness was comparable or even better than TSA [201]. Reduced fibrotic scars and fatty infiltration, as well as a decreased inflammatory infiltrate, were observed in the muscles of *mdx* mice exposed to 5 and 10 mg/kg/day of givinostat whereas a dose of 5 mg/kg/day led to a significantly better muscle performance assessed by an exhaustion treadmill test [201]. These data suggested the possible therapeutic options for this compound in humans; therefore, a phase 1 clinical trial was started in January 2013 (NCT01761292). As summarized by Bettica et al. [205], 20 DMD boys aged 7–10 years on stable corticosteroid treatment were treated for ≥ 12 months with givinostat (25 mg BID to 37.5 mg BID, depending on the scheme). Although a functional benefit was not observed in this study (which may be related to the small sample size), the histological evaluation of muscle biopsy showed an increase in muscle fiber size, reduction of fibrosis, tissue necrosis, and fatty replacement. Givinostat has been granted an orphan drug designation (EU/3/12/1009) by the EMA for the treatment of DMD and the ongoing phase 3 trials, with an estimated enrollment of 313 participants (100 for NCT03373968 and 213 for NCT02851797), are underway and will hopefully bring more data about the effectiveness of HDACi and the potential of long-term treatment in DMD patients.

### The upregulation of utrophin by drug therapy is a plausible therapeutic approach in the treatment of DMD

As mentioned earlier, gene therapy aimed at utrophin overexpression represents a valuable therapeutic strategy. Pharmacological factors have been found to upregulate utrophin expression and act protectively in animal models of DMD. One of such utrophin modulator, ezutromid (SMT C1100), has been shown to be safe for healthy adult men [63]. Interestingly, recent results from the phase 1b trial have underlined the importance of a proper, balanced diet and the role of milk supplementation for the better absorption of ezutromid (as it is highly insoluble in water), resulting in higher systemic exposure in DMD patients [206]. However, based on the information from clinicaltrial.gov, a phase 2 trial with this compound (NCT02858362) was terminated due to a lack of efficacy (Table 1). Summit Therapeutics has developed other molecules (but belonging to the same series as ezutromid) with protective effects demonstrated in *mdx* mice [207] and other compounds like nabumetone, which can modulate utrophin promoter, have been tested in vitro [208].

Utrophin modulation can be a promising therapeutic strategy for all DMD patients irrespective of their dystrophin mutation. However, although a milder disease in *mdx* mice expressing utrophin suggests the crucial protective role of this protein, in DMD patients, who have naturally upregulated the expression of utrophin, the diseases progress as well [18]. This might be caused by the fact that utrophin is not able to replace all symptoms of dystrophin deficiency as it lacks NO-binding site [68] and is not able to prevent functional ischemia during muscle contraction [69].

### Regulation of cardiac dysfunction and hypoxia prevention may have an impact on DMD progression

DMD patients could benefit from heart-targeted therapies [191–193]. Angiotensin-converting enzyme inhibitors (ACEIs) may exert cardioprotective effects in DMD individuals [209] as angiotensin II acts as a destructive agent, promoting oxidative stress, heart fibrosis, and cardiomyocyte death [210]. A 10-year follow-up study revealed a significantly higher survival rate after presymptomatic treatment with perindopril (ACEI) for 3 years [209]. It is recommended to start therapy with ACEIs even before the onset of LV dysfunction [18]. Also, angiotensin receptor blockers (like losartan) can be used as an alternative to ACEIs to suppress the deleterious effects of angiotensin in heart failure [211]. Other drugs, used mostly as the second-line therapy in DMD patients already presenting signs of LV dysfunction, are beta-adrenergic receptors (β-AR) blockers [212–214]. Mineralocorticoid receptor (MR) antagonists such as eplerenone, which have not only cardioprotective but also anti-fibrotic effects, have also been considered [215] (Fig. 2).

A lack of dystrophin leads to mislocalization of nNOS, a part of the DGC complex, at the sarcolemma and subsequent reduction in nNOS mRNA and protein level [5], nNOS activity [6], and NO production [7] causing unbalanced muscle oxygenation and a lack of protective effect against excessive sympathetic vasoconstriction and leading to functional muscle ischemia when the dystrophic muscles are exercised [216]. The decrease in nNOS activity results in the downregulation of the activity of soluble guanyl cyclase (sGC) and decreased cGMP level. Therefore, factors inhibiting cGMP phosphodiesterase (PDE5) activity thereby prolonging the biological half-life of cGMP [217] may be of particular interest in the context of DMD treatment. Of note, inhibition of PDE5 activity (by, for example, sildenafil or tadalafil) was shown to alleviate the DMD phenotype, mostly through the reduction of fibrosis [218] as well as the cardioprotective effects both in *mdx* mice and in the dog model [219]. Nelson et al. [220] have checked the effect of PDE5 inhibition on muscle ischemia and skeletal muscle blood flow. Research performed on ten DMD patients (receiving background therapy with GCs), compared to perfectly matched ten control male individuals showed attenuation of muscle ischemia and impaired functional sympatholysis by PDE5 inhibition [220]. However, a bigger study, performed on more than 300 DMD patients divided into control and tadalafil-treated groups, revealed no effect of PDE inhibitor given in two different regimens in comparison to placebo-treated boys (all taking standard GCs) [221]. This controlled phase 3 trial assessing the possible functional improvement (e.g., by measuring 6MWD and other motor functions) after once-daily supplementation with PDE5 inhibitor for 48 weeks failed to show its protective effect (Table 1, NCT01865084). Further studies are needed to fully assess the possible positive outcome of tadalafil on both cardiac and leg muscle injury as several factors (including the age of patients, a scheme of exercise performed by the affected patients) may influence the final conclusions.

Pharmacological treatments have demonstrated some beneficial effects, but it is still not fully known if prophylactic cardiac therapy should be applied to all DMD patients and/or what kind of combination of the cardioprotective drug should be used.

## New modulators of DMD progression

### Targeting angiogenesis in DMD

DMD is not only a muscle-specific disease, as dystrophin is also expressed in other cell types, including vascular smooth muscle cells and ECs [22, 222]. This suggests that blood vessel formation in DMD patients and the properties of these cells might be impaired.

Although contrasting data have been published regarding the status of angiogenesis in *mdx* mice [23, 223], these data might be very much affected by, among others, the age of animals, as reviewed by us recently [8]. We have found diminished expression of angiogenic factors in adult *mdx* mice, including vascular endothelial growth factor (VEGF) and CD31 (Pecam1) [224]. On the other hand, it was demonstrated that the overexpression of pro-angiogenic and pro-myogenic VEGF in *mdx* mice led to a reduction of necrosis, increased regenerating fibers, and capillary density in the regenerating area [225]. Inhibition of VEGFR-1, a negative regulator of angiogenesis, overexpression of pro-angiogenic angiopoietin-1 (Ang1), or combined VEGF/Ang1 delivery has also been considered therapeutic possibilities (reviewed in [8]).

### The role of heme oxygenase-1 in DMD

Heme oxygenase-1 (HO-1, encoded by *Hmox1* gene) is a cytoprotective enzyme that not only degrades heme to carbon monoxide (CO), ferrous iron (inducing the synthesis of protective ferritin) and biliverdin (subsequently converted into antioxidant bilirubin by biliverdin reductase), but also possesses pleiotropic, non-canonical functions (for review, see: [226–228]). HO-1 exerts antioxidant effects, regulates apoptosis and autophagy, and has anti-inflammatory properties and pro-angiogenic functions (for review see: [226–228]). We have demonstrated the involvement of HO-1 in VEGF [229, 230] and SDF-1-dependent angiogenesis [231], and its contribution to angiogenesis-related processes like tumorigenesis [232–235] and wound healing [236].

Recent studies performed by our group revealed the contribution of HO-1 to DMD onset and progression. First, we have found that HO-1 plays an important role in muscle (progenitor) cell biology. HO-1 may promote the proliferation of myoblasts and muscle regeneration when the short-term expression is evaluated [237]; however, long-term expression leads to decreased differentiation through the modulation of muscle-specific microRNAs (myomirs) [238]. In both acute (cardiotoxin (CTX)-induced) [239] and chronic (*mdx* mice) [240] muscle damage models, a lack of HO-1 accelerated the pathological situation. Increased fibrosis, inflammation, muscle functioning (as demonstrated by the impaired running capacity), and disturbed and enhanced differentiation of SCs were evident in dystrophic mice with knock-out of HO-1 [240].

HO-1 also acts as an antioxidant factor. Antioxidants have been suggested as a potential treatment for DMD patients. Promising results were obtained when N-acetylcysteine (NAC) was evaluated in the mouse model of the disease [241, 242]; however, recent work by Pinniger et al. [243] revealed some adverse effects (reduced body, liver, and muscle weight) of this treatment. Other studies with green tea extract or epigallocatechin-3-gallate (EGCG) known to exert antioxidant properties showed reduced necrotic fibers and increased resistance to fatigue of dystrophic muscles [244, 245]. However, no beneficial outcome for patients treated with vitamin B, vitamin E, or penicillamine was observed in studies performed in the 1960s–1980s [246–248]. Generally, the results of clinical trials with antioxidants are rather disappointing (not only in the context of DMD) which may be caused by a difficulty in the choice of appropriate dose/regimen of antioxidant treatment as well as many signaling pathways that are affected by non-specific factors [249].

### Can statins be beneficial in DMD patients?

In 2015, Whitehead et al. [250] described the beneficial effects of statins in *mdx* mice. These findings were somehow surprising, as statins, 3-hydroxy-3-methylglutaryl coenzyme A (HMG-CoA) reductase inhibitors, and drugs widely used for the treatment of hypercholesterolemia and reduction of atherosclerosis (reviewed in [251]) were mostly considered as compounds inducing skeletal muscle myopathy and rhabdomyolysis [252, 253].

In contrast to these results, in studies performed by Whitehead et al., statins did not elicit any devastating effects on muscle function. On the contrary, decreased inflammation, fibrosis, and oxidative stress and accelerated muscle force were evident after a moderate daily dose of simvastatin [250]. Moreover, simvastatin treatment attenuated impaired autophagy which is known to contribute to DMD progression [254].

Statins are known to regulate angiogenesis [255–257] and exert cardioprotective effects [258]. Therefore, it is not surprising that their effect on cardiac complications in *mdx* mice was also evaluated. In addition to having protective effects on skeletal muscles, simvastatin also improved heart functions [259]. In older *mdx* mice (12 months old) spontaneously developing cardiomyopathy, simvastatin significantly enhanced diastolic function as evidenced by echocardiography and it also halted myocardial fibrosis. Improvement in skeletal and cardiac muscle physiological functions by statins in *mdx* mice makes these drugs good candidates for clinical evaluation in DMD patients.

The results obtained by Whitehead et al. [250] are in contrast to the above-mentioned report about statin-induced myopathy. However, when a meta-analysis comparing the risk of the toxic statin-related muscle effect was performed, there was no difference when compared to patients treated with placebo [260]. The explanation for this discrepancy may be that statins are mostly prescribed to older patients (whereas DMD is a disease typically affecting very young boys) and the adverse effects of these drugs are aggravated by several factors, including exercise and female sex [261, 262], both not relevant to DMD boys.

### MicroRNAs in DMD pathology

Non-coding miRNAs target roughly 60% of human genes, emphasising their essential role in the regulation of many biological processes [263]. We have shown the contribution of numerous miRNAs in the differentiation of murine myoblast cells and SCs, and as mentioned earlier, we have examined their impact on muscle regeneration in a model of CTX-induced skeletal muscle injury and *mdx* mice [238, 239].

A detailed analysis of the role of specific microRNAs in DMD revealed miR-200c to be responsible for muscle wasting and myotube loss, most probably via a p66Shc-dependent mechanism, increased ROS production, and oxidative stress acceleration [264]. Interestingly, our recent data [265] suggest that inhibition of miR-378, recognized as an important mediator of differentiation and proliferation of myoblasts [266–268] and a regulator of skeletal muscle vascularization [269], may attenuate dystrophic phenotype. On the other hand, we have found that miR-146a, known to diminish inflammation and fibrosis in different tissues by downregulating the expression of proinflammatory cytokines, does not significantly affect the deleterious pathological events of DMD progression in *mdx* mice [224]. Another study pointed out the lack of beneficial effect of miR-92a inhibition on DMD progression in *mdx* mice despite the increased capillary density and tissue perfusion that was found after antagomir-92a [270].

### Immunotherapy might help treat DMD

Recent studies by Zschüntzsch et al. [271, 272] provide evidence that human immunoglobulin G (IgG) treatment can improve DMD outcomes. The first trial analyzing the effects of 8-week i.p. injections of IgG on muscle performance [271] was then repeated to see the long-term effectiveness and also to analyse the possible benefits for heart degeneration [272]. In the second study, 3-week-old *mdx* mice following antibody treatment (IgG 2 g/kg body weight, administered monthly over 18 months by i.p. injection) had a lower serum level of CK as well as decreased expression of inflammatory markers and a significant reduction of the infiltration of T cells to various muscles in comparison to NaCl solution-treated controls. Moreover, the running performance was better in IgG-injected animals. In addition, the cardiac phenotype was mitigated—fractional area shortening was improved, whereas cardiac fibrosis and the infiltration of T cells were decreased. This study in the mouse model is quite preliminary, but it may open a new direction for the treatment of DMD.

## Future perspectives

Although numerous different targets/strategies and even clinical trials have been performed, DMD is still an incurable disease. Many questions have to be solved, including ones about the level of dystrophin necessary to have a positive effect on stopping DMD progression and especially the misleading information based on the method used. Moreover, both in the case of gene delivery and exon skipping approaches, the effectiveness of dystrophin restoration in the heart muscle is the greatest problem. There is even an apprehension that effective therapy in skeletal muscles, resulting in improved patient mobility, can increase the heart overload and actually worsen conditions [82]. Therefore, although restoration of dystrophin expression is the obvious aim to treat the disease’s primary cause, other approaches to modulate the severity of the disease can be helpful. One can presume that the early detection of heart involvement may lead to introducing therapies which, like GCs, can significantly postpone disease progression and improve the patient’s life. Therefore, there is an urgent need for better diagnosis of cardiac involvement in DMD patients. More importantly, the systemic markers routinely used for assessing heart failure and cardiac damage, namely the increased serum brain natriuretic peptide (BNP) and troponin, are not good predictors in DMD patients, as their levels may not be significantly altered even in the case of heart damage [273]. Therefore, for the future, the identification of new, more specific markers is undeniably needed. Finally, more attention should be placed on the use of nutraceuticals and dietary supplements in DMD patients. Although vitamin D, taurine, creatine, curcumin, etc. can potentially attenuate inflammation, oxidative stress, and other pathological processes during disease progression, they could also exert harmful effects either in high doses or when cross-reacting with other drugs which the patient is taking [274, 275].

We hope that more research experiments, including our attempts, and clinical trials will help in providing new therapeutic approaches, better patient selection, and stratification for future trials.

## References

[1] Carter JC, Sheehan DW, Prochoroff A, Birnkrant DJ (2018). Muscular dystrophies. Clin Chest Med.

[2] Bladen CL, Salgado D, Monges S, Foncuberta ME, Kekou K, Kosma K (2015). The TREAT-NMD DMD ss: analysis of more than 7000 Duchenne muscular dystrophy mutations. Hum Mutat.

[3] Hoffman EP, Brown RH, Kunkel LM (1987). Dystrophin: the protein product of the Duchenne muscular dystrophy locus. Cell.

[4] Gillis JM (1996). Membrane abnormalities and Ca homeostasis in muscles of the mdx mouse, an animal model of the Duchenne muscular dystrophy: a review. Acta Physiol Scand.

[5] Vaghy PL, Fang J, Wu W, Vaghy LP (1998). Increased caveolin-3 levels in mdx mouse muscles. FEBS Lett.

[6] Chang WJ, Iannaccone ST, Lau KS, Masters BS, McCabe TJ, McMillan K (1996). Neuronal nitric oxide synthase and dystrophin-deficient muscular dystrophy. Proc Natl Acad Sci USA.

[7] Kasai T, Abeyama K, Hashiguchi T, Fukunaga H, Osame M, Maruyama I (2004). Decreased total nitric oxide production in patients with duchenne muscular dystrophy. J Biomed Sci.

[8] Podkalicka P, Mucha O, Dulak J, Loboda A (2019). Targeting angiogenesis in Duchenne muscular dystrophy. Cell Mol Life Sci.

[9] Yucel N, Chang AC, Day JW, Rosenthal N, Blau HM (2018). Humanizing the mdx mouse model of DMD: the long and the short of it. NPJ Regen Med.

[10] Bulfield G, Siller WG, Wight PA, Moore KJ (1984). X chromosome-linked muscular dystrophy (mdx) in the mouse. Proc Natl Acad Sci USA.

[11] Ryder-Cook AS, Sicinski P, Thomas K, Davies KE, Worton RG, Barnard EA (1988). Localization of the mdx mutation within the mouse dystrophin gene. EMBO J.

[12] Kornegay JN (2017). The golden retriever model of Duchenne muscular dystrophy. Skelet Muscle.

[13] Brinkmeyer-Langford C, Chu C, Balog-Alvarez C, Yu X, Cai JJ, Nabity M (2018). Expression profiling of disease progression in canine model of Duchenne muscular dystrophy. PLoS One.

[14] Birnkrant DJ, Bushby K, Bann CM, Apkon SD, Blackwell A, Brumbaugh D (2018). Diagnosis and management of Duchenne muscular dystrophy, part 1: diagnosis, and neuromuscular, rehabilitation, endocrine, and gastrointestinal and nutritional management. Lancet Neurol.

[15] Szigyarto CA-K, Spitali P (2018). Biomarkers of Duchenne muscular dystrophy: current findings. Degener Neurol Neuromuscul Dis.

[16] Anaya-Segura MA, García-Martínez FA, Montes-Almanza LA, Díaz B-G, Avila-Ramírez G, Alvarez-Maya I (2015). Non-invasive biomarkers for Duchenne muscular dystrophy and carrier detection. Molecules.

[17] Emery AEH (2002). The muscular dystrophies. Lancet.

[18] Kamdar F, Garry DJ (2016). Dystrophin-deficient cardiomyopathy. J Am Coll Cardiol.

[19] Nigro G, Comi LI, Politano L, Bain RJ (1990). The incidence and evolution of cardiomyopathy in Duchenne muscular dystrophy. Int J Cardiol.

[20] Nguyen Q, Yokota T (2019). Antisense oligonucleotides for the treatment of cardiomyopathy in Duchenne muscular dystrophy. Am J Transl Res.

[21] van Westering TLE, Betts CA, Wood MJA (2015). Current understanding of molecular pathology and treatment of cardiomyopathy in duchenne muscular dystrophy. Molecules.

[22] Loufrani L, Matrougui K, Gorny D, Duriez M, Blanc I, Lévy BI (2001). Flow (shear stress)-induced endothelium-dependent dilation is altered in mice lacking the gene encoding for dystrophin. Circulation.

[23] Palladino M, Gatto I, Neri V, Straino S, Smith RC, Silver M (2013). Angiogenic impairment of the vascular endothelium: a novel mechanism and potential therapeutic target in muscular dystrophy. Arterioscler Thromb Vasc Biol.

[24] Loufrani L, Dubroca C, You D, Li Z, Levy B, Paulin D (2004). Absence of dystrophin in mice reduces NO-dependent vascular function and vascular density: total recovery after a treatment with the aminoglycoside gentamicin. Arterioscler Thromb Vasc Biol.

[25] Hugnot JP, Gilgenkrantz H, Chafey P, Lambert M, Eveno E, Kaplan JC (1993). Expression of the dystrophin gene in cultured fibroblasts. Biochem Biophys Res Commun.

[26] D’Amario D, Amodeo A, Adorisio R, Tiziano FD, Leone AM, Perri G (2017). A current approach to heart failure in Duchenne muscular dystrophy. Heart.

[27] Rafael-Fortney JA, Chadwick JA, Raman SV (2016). Duchenne muscular dystrophy mice and men: can understanding a genetic cardiomyopathy inform treatment of other myocardial diseases?. Circ Res.

[28] McNally EM, Kaltman JR, Benson DW, Canter CE, Cripe LH, Duan D (2015). Contemporary cardiac issues in Duchenne muscular dystrophy. Working Group of the National Heart, Lung, and Blood Institute in collaboration with Parent Project Muscular Dystrophy. Circulation.

[29] Amodeo A, Adorisio R (2012). Left ventricular assist device in Duchenne cardiomyopathy: can we change the natural history of cardiac disease?. Int J Cardiol.

[30] Iodice F, Testa G, Averardi M, Brancaccio G, Amodeo A, Cogo P (2015). Implantation of a left ventricular assist device as a destination therapy in Duchenne muscular dystrophy patients with end stage cardiac failure: management and lessons learned. Neuromuscul Disord.

[31] Miller LW, Rogers JG (2018). Evolution of left ventricular assist device therapy for advanced heart failure: a review. JAMA Cardiol.

[32] Tyler KL (2003). Origins and early descriptions of “Duchenne muscular dystrophy”. Muscle Nerve.

[33] Goyenvalle A, Seto JT, Davies KE, Chamberlain J (2011). Therapeutic approaches to muscular dystrophy. Hum Mol Genet.

[34] Mendell JR, Campbell K, Rodino-Klapac L, Sahenk Z, Shilling C, Lewis S (2010). Dystrophin immunity in Duchenne’s muscular dystrophy. N Engl J Med.

[35] Chamberlain JS. Dystrophin Levels Required for Genetic Correction of Duchenne Muscular Dystrophy (n.d.)

[36] Yue Y, Skimming JW, Liu M, Strawn T, Duan D (2004). Full-length dystrophin expression in half of the heart cells ameliorates beta-isoproterenol-induced cardiomyopathy in mdx mice. Hum Mol Genet.

[37] Neri M, Torelli S, Brown S, Ugo I, Sabatelli P, Merlini L (2007). Dystrophin levels as low as 30% are sufficient to avoid muscular dystrophy in the human. Neuromuscul Disord.

[38] Aartsma-Rus A, Morgan J, Lonkar P, Neubert H, Owens J, Binks M (2019). Report of a TREAT-NMD/World Duchenne Organisation Meeting on Dystrophin Quantification Methodology. J Neuromuscul Dis.

[39] Aguti S, Malerba A, Zhou H (2018). The progress of AAV-mediated gene therapy in neuromuscular disorders. Expert Opin Biol Ther.

[40] Wasala LP, Hakim CH, Yue Y, Yang NN, Duan D (2019). Systemic delivery of adeno-associated viral vectors in mice and dogs. Methods Mol Biol.

[41] Inagaki K, Fuess S, Storm TA, Gibson GA, Mctiernan CF, Kay MA (2006). Robust systemic transduction with AAV9 vectors in mice: efficient global cardiac gene transfer superior to that of AAV8. Mol Ther.

[42] Chamberlain JR, Chamberlain JS (2017). Progress toward gene therapy for Duchenne muscular dystrophy. Mol Ther.

[43] Yuasa K, Miyagoe Y, Yamamoto K, Nabeshima Y, Dickson G, Takeda S (1998). Effective restoration of dystrophin-associated proteins in vivo by adenovirus-mediated transfer of truncated dystrophin cDNAs. FEBS Lett.

[44] Koenig M, Beggs AH, Moyer M, Scherpf S, Heindrich K, Bettecken T (1989). The molecular basis for Duchenne versus Becker muscular dystrophy: correlation of severity with type of deletion. Am J Hum Genet.

[45] Harper SQ, Hauser MA, DelloRusso C, Duan D, Crawford RW, Phelps SF (2002). Modular flexibility of dystrophin: implications for gene therapy of Duchenne muscular dystrophy. Nat Med.

[46] Sakamoto M, Yuasa K, Yoshimura M, Yokota T, Ikemoto T, Suzuki M (2002). Micro-dystrophin cDNA ameliorates dystrophic phenotypes when introduced into mdx mice as a transgene. Biochem Biophys Res Commun.

[47] Duan D, Systemic AAV (2018). Micro-dystrophin gene therapy for Duchenne muscular dystrophy. Mol Ther.

[48] Mendell JR, Sahenk Z, Lehman K, Nease C, Lowes LP, Miller NF (2020). Assessment of systemic delivery of rAAVrh74.MHCK7.micro-dystrophin in children with Duchenne muscular dystrophy: a nonrandomized controlled trial. JAMA Neurol.

[49] Lai Y, Thomas GD, Yue Y, Yang HT, Li D, Long C (2009). Dystrophins carrying spectrin-like repeats 16 and 17 anchor nNOS to the sarcolemma and enhance exercise performance in a mouse model of muscular dystrophy. J Clin Investig.

[50] Hakim CH, Wasala NB, Pan X, Kodippili K, Yue Y, Zhang K (2017). A five-repeat micro-dystrophin gene ameliorated dystrophic phenotype in the severe DBA/2J-mdx model of duchenne muscular dystrophy. Mol Ther Methods Clin Dev.

[51] Ramos JN, Hollinger K, Bengtsson NE, Allen JM, Hauschka SD, Chamberlain JS (2019). Development of novel micro-dystrophins with enhanced functionality. Mol Ther.

[52] Pfizer’s New Phase 1b Results of Gene Therapy in Ambulatory Boys with Duchenne Muscular Dystrophy (DMD) Support Advancement into Pivotal Phase 3 Study n.d. https://investors.pfizer.com/investor-news/press-release-details/2020/Pfizers-New-Phase-1b-Results-of-Gene-Therapy-in-Ambulatory-Boys-with-Duchenne-Muscular-Dystrophy-DMD-Support-Advancement-into-Pivotal-Phase-3-Study/default.aspx. Accessed 20 May 2020.

[53] Letter to the Duchenne Community: Update on SGT-001 Phase I/II Clinical Hold for IGNITE DMD Program. Solid Biosciences n.d. https://www.solidbio.com/about/media/news/letter-to-the-duchenne-community-update-on-sgt-001-phase-i-ii-clinical-hold-for-ignite-dmd-program. Accessed 20 May 2020.

[54] Vila MC, Novak JS, Benny Klimek M, Li N, Morales M, Fritz AG (2019). Morpholino-induced exon skipping stimulates cell-mediated and humoral responses to dystrophin in mdx mice. J Pathol.

[55] Love DR, Hill DF, Dickson G, Spurr NK, Byth BC, Marsden RF (1989). An autosomal transcript in skeletal muscle with homology to dystrophin. Nature.

[56] Schofield J, Houzelstein D, Davies K, Buckingham M, Edwards YH (1993). Expression of the dystrophin-related protein (utrophin) gene during mouse embryogenesis. Dev Dyn.

[57] Clerk A, Morris GE, Dubowitz V, Davies KE, Sewry CA (1993). Dystrophin-related protein, utrophin, in normal and dystrophic human fetal skeletal muscle. Histochem J.

[58] Weir AP, Burton EA, Harrod G, Davies KE (2002). A- and B-utrophin have different expression patterns and are differentially up-regulated in mdx muscle. J Biol Chem.

[59] Nguyen TM, Ellis JM, Love DR, Davies KE, Gatter KC, Dickson G (1991). Localization of the DMDL gene-encoded dystrophin-related protein using a panel of nineteen monoclonal antibodies: presence at neuromuscular junctions, in the sarcolemma of dystrophic skeletal muscle, in vascular and other smooth muscles, and in proliferating brain cell lines. J Cell Biol.

[60] Helliwell TR, Man NT, Morris GE, Davies KE (1992). The dystrophin-related protein, utrophin, is expressed on the sarcolemma of regenerating human skeletal muscle fibres in dystrophies and inflammatory myopathies. Neuromuscul Disord.

[61] Deconinck AE, Rafael JA, Skinner JA, Brown SC, Potter AC, Metzinger L (1997). Utrophin-dystrophin-deficient mice as a model for Duchenne muscular dystrophy. Cell.

[62] Grady RM, Teng H, Nichol MC, Cunningham JC, Wilkinson RS, Sanes JR (1997). Skeletal and cardiac myopathies in mice lacking utrophin and dystrophin: a model for Duchenne muscular dystrophy. Cell.

[63] Tinsley J, Deconinck N, Fisher R, Kahn D, Phelps S, Gillis JM (1998). Expression of full-length utrophin prevents muscular dystrophy in mdx mice. Nat Med.

[64] Kennedy TL, Moir L, Hemming S, Edwards B, Squire S, Davies K (2017). Utrophin influences mitochondrial pathology and oxidative stress in dystrophic muscle. Skelet Muscle.

[65] Mishra MK, Loro E, Sengupta K, Wilton SD, Khurana TS (2017). Functional improvement of dystrophic muscle by repression of utrophin: let-7c interaction. PLoS One.

[66] Kornegay JN, Li J, Bogan JR, Bogan DJ, Chen C, Zheng H (2010). Widespread muscle expression of an AAV9 human mini-dystrophin vector after intravenous injection in neonatal dystrophin-deficient dogs. Mol Ther.

[67] Song Y, Morales L, Malik AS, Mead AF, Greer CD, Mitchell MA (2019). Non-immunogenic utrophin gene therapy for the treatment of muscular dystrophy animal models. Nat Med.

[68] Li D, Bareja A, Judge L, Yue Y, Lai Y, Fairclough R (2010). Sarcolemmal nNOS anchoring reveals a qualitative difference between dystrophin and utrophin. J Cell Sci.

[69] Belanto JJ, Mader TL, Eckhoff MD, Strandjord DM, Banks GB, Gardner MK (2014). Microtubule binding distinguishes dystrophin from utrophin. PNAS.

[70] Guiraud S, Edwards B, Babbs A, Squire SE, Berg A, Moir L (2019). The potential of utrophin and dystrophin combination therapies for Duchenne muscular dystrophy. Hum Mol Genet.

[71] Dowling JJ (2016). Eteplirsen therapy for Duchenne muscular dystrophy: skipping to the front of the line. Nat Rev Neurol.

[72] Malhotra SB, Hart KA, Klamut HJ, Thomas NS, Bodrug SE, Burghes AH (1988). Frame-shift deletions in patients with Duchenne and Becker muscular dystrophy. Science.

[73] Verma A (2018). Recent advances in antisense oligonucleotide therapy in genetic neuromuscular diseases. Ann Indian Acad Neurol.

[74] Niks EH, Aartsma-Rus A (2017). Exon skipping: a first in class strategy for Duchenne muscular dystrophy. Expert Opin Biol Ther.

[75] Cirak S, Arechavala-Gomeza V, Guglieri M, Feng L, Torelli S, Anthony K (2011). Exon skipping and dystrophin restoration in patients with Duchenne muscular dystrophy after systemic phosphorodiamidate morpholino oligomer treatment: an open-label, phase 2, dose-escalation study. Lancet.

[76] Goemans NM, Tulinius M, van den Akker JT, Burm BE, Ekhart PF, Heuvelmans N (2011). Systemic administration of PRO051 in Duchenne’s muscular dystrophy. N Engl J Med.

[77] van Deutekom JCT, van Ommen GJ-B (2003). Advances in Duchenne muscular dystrophy gene therapy. Nat Rev Genet.

[78] Lim KRQ, Maruyama R, Yokota T (2017). Eteplirsen in the treatment of Duchenne muscular dystrophy. Drug Des Devel Ther.

[79] Kesselheim AS, Avorn J (2016). Approving a problematic muscular dystrophy drug: implications for FDA policy. JAMA.

[80] Viltolarsen DS (2020). First approval. Drugs.

[81] Alter J, Lou F, Rabinowitz A, Yin H, Rosenfeld J, Wilton SD (2006). Systemic delivery of morpholino oligonucleotide restores dystrophin expression bodywide and improves dystrophic pathology. Nat Med.

[82] Townsend D, Yasuda S, Li S, Chamberlain JS, Metzger JM (2008). Emergent dilated cardiomyopathy caused by targeted repair of dystrophic skeletal muscle. Mol Ther.

[83] Yin H, Moulton HM, Seow Y, Boyd C, Boutilier J, Iverson P (2008). Cell-penetrating peptide-conjugated antisense oligonucleotides restore systemic muscle and cardiac dystrophin expression and function. Hum Mol Genet.

[84] Betts C, Saleh AF, Arzumanov AA, Hammond SM, Godfrey C, Coursindel T (2012). Pip6-PMO, A new generation of peptide-oligonucleotide conjugates with improved cardiac exon skipping activity for DMD treatment. Mol Ther Nucleic Acids.

[85] Echigoya Y, Yokota T (2014). Skipping multiple exons of dystrophin transcripts using cocktail antisense oligonucleotides. Nucleic Acid Ther.

[86] Aslesh T, Maruyama R, Yokota T (2018). Skipping multiple exons to treat DMD-promises and challenges. Biomedicines.

[87] Miskew Nichols B, Aoki Y, Kuraoka M, Lee JJA, Takeda S, Yokota T (2016). Multi-exon skipping using cocktail antisense oligonucleotides in the canine X-linked muscular dystrophy. J Vis Exp.

[88] Echigoya Y, Nakamura A, Nagata T, Urasawa N, Lim KRQ, Trieu N (2017). Effects of systemic multiexon skipping with peptide-conjugated morpholinos in the heart of a dog model of Duchenne muscular dystrophy. Proc Natl Acad Sci USA.

[89] Howard MT, Shirts BH, Petros LM, Flanigan KM, Gesteland RF, Atkins JF (2000). Sequence specificity of aminoglycoside-induced stop condon readthrough: potential implications for treatment of Duchenne muscular dystrophy. Ann Neurol.

[90] Dunant P, Walter MC, Karpati G, Lochmüller H (2003). Gentamicin fails to increase dystrophin expression in dystrophin-deficient muscle. Muscle Nerve.

[91] Barton-Davis ER, Cordier L, Shoturma DI, Leland SE, Sweeney HL (1999). Aminoglycoside antibiotics restore dystrophin function to skeletal muscles of mdx mice. J Clin Investig.

[92] Malik V, Rodino-Klapac LR, Viollet L, Wall C, King W, Al-Dahhak R (2010). Gentamicin-induced readthrough of stop codons in Duchenne muscular dystrophy. Ann Neurol.

[93] Guan MX, Fischel-Ghodsian N, Attardi G (2000). A biochemical basis for the inherited susceptibility to aminoglycoside ototoxicity. Hum Mol Genet.

[94] Nudelman I, Glikin D, Smolkin B, Hainrichson M, Belakhov V, Baasov T (2010). Repairing faulty genes by aminoglycosides: development of new derivatives of geneticin (G418) with enhanced suppression of diseases-causing nonsense mutations. Bioorg Med Chem.

[95] Bushby K, Finkel R, Wong B, Barohn R, Campbell C, Comi GP (2014). Ataluren treatment of patients with nonsense mutation dystrophinopathy. Muscle Nerve.

[96] Finkel RS, Flanigan KM, Wong B, Bönnemann C, Sampson J, Sweeney HL (2013). Phase 2a study of ataluren-mediated dystrophin production in patients with nonsense mutation Duchenne muscular dystrophy. PLoS One.

[97] McDonald CM, Campbell C, Torricelli RE, Finkel RS, Flanigan KM, Goemans N (2017). Ataluren in patients with nonsense mutation Duchenne muscular dystrophy (ACT DMD): a multicentre, randomised, double-blind, placebo-controlled, phase 3 trial. Lancet.

[98] Nakamura A (2019). Mutation-based therapeutic strategies for duchenne muscular dystrophy: from genetic diagnosis to therapy. J Pers Med.

[99] Young CS, Hicks MR, Ermolova NV, Nakano H, Jan M, Younesi S (2016). A single CRISPR-Cas9 deletion strategy that targets the majority of DMD patients restores dystrophin function in hiPSC-derived muscle cells. Cell Stem Cell.

[100] Min Y-L, Bassel-Duby R, Olson EN (2019). CRISPR correction of Duchenne muscular dystrophy. Annu Rev Med.

[101] Zhang Y, Long C, Bassel-Duby R, Olson EN (2018). Myoediting: toward prevention of muscular dystrophy by therapeutic genome editing. Physiol Rev.

[102] Long C, McAnally JR, Shelton JM, Mireault AA, Bassel-Duby R, Olson EN (2014). Prevention of muscular dystrophy in mice by CRISPR/Cas9-mediated editing of germline DNA. Science.

[103] Nelson CE, Hakim CH, Ousterout DG, Thakore PI, Moreb EA, Castellanos Rivera RM (2016). In vivo genome editing improves muscle function in a mouse model of Duchenne muscular dystrophy. Science.

[104] Tabebordbar M, Zhu K, Cheng JKW, Chew WL, Widrick JJ, Yan WX (2016). In vivo gene editing in dystrophic mouse muscle and muscle stem cells. Science.

[105] Long C, Amoasii L, Mireault AA, McAnally JR, Li H, Sanchez-Ortiz E (2016). Postnatal genome editing partially restores dystrophin expression in a mouse model of muscular dystrophy. Science.

[106] Amoasii L, Hildyard JCW, Li H, Sanchez-Ortiz E, Mireault A, Caballero D (2018). Gene editing restores dystrophin expression in a canine model of Duchenne muscular dystrophy. Science.

[107] Moretti A, Fonteyne L, Giesert F, Hoppmann P, Meier AB, Bozoglu T (2020). Somatic gene editing ameliorates skeletal and cardiac muscle failure in pig and human models of Duchenne muscular dystrophy. Nat Med.

[108] El Refaey M, Xu L, Gao Y, Canan BD, Adesanya TMA, Warner SC (2017). In vivo genome editing restores dystrophin expression and cardiac function in dystrophic mice. Circ Res.

[109] Kimberland ML, Hou W, Alfonso-Pecchio A, Wilson S, Rao Y, Zhang S (2018). Strategies for controlling CRISPR/Cas9 off-target effects and biological variations in mammalian genome editing experiments. J Biotechnol.

[110] Zhang X-H, Tee LY, Wang X-G, Huang Q-S, Yang S-H (2015). Off-target effects in CRISPR/Cas9-mediated genome engineering. Mol Ther Nucleic Acids.

[111] Dumonceaux J, Marie S, Beley C, Trollet C, Vignaud A, Ferry A (2010). Combination of myostatin pathway interference and dystrophin rescue enhances tetanic and specific force in dystrophic mdx mice. Mol Ther.

[112] Hoogaars WMH, Mouisel E, Pasternack A, Hulmi JJ, Relizani K, Schuelke M (2012). Combined effect of AAV-U7-induced dystrophin exon skipping and soluble activin type IIB receptor in mdx mice. Hum Gene Ther.

[113] Amthor H, Hoogaars WMH (2012). Interference with myostatin/ActRIIB signaling as a therapeutic strategy for Duchenne muscular dystrophy. Curr Gene Ther.

[114] Garber K (2016). No longer going to waste. Nat Biotechnol.

[115] Wagner KR, Fleckenstein JL, Amato AA, Barohn RJ, Bushby K, Escolar DM (2008). A phase I/IItrial of MYO-029 in adult subjects with muscular dystrophy. Ann Neurol.

[116] Campbell C, McMillan HJ, Mah JK, Tarnopolsky M, Selby K, McClure T (2017). Myostatin inhibitor ACE-031 treatment of ambulatory boys with Duchenne muscular dystrophy: results of a randomized, placebo-controlled clinical trial. Muscle Nerve.

[117] Mariot V, Joubert R, Hourdé C, Féasson L, Hanna M, Muntoni F (2017). Downregulation of myostatin pathway in neuromuscular diseases may explain challenges of anti-myostatin therapeutic approaches. Nat Commun.

[118] Burch PM, Pogoryelova O, Palandra J, Goldstein R, Bennett D, Fitz L (2017). Reduced serum myostatin concentrations associated with genetic muscle disease progression. J Neurol.

[119] Lu-Nguyen NB, Jarmin SA, Saleh AF, Popplewell L, Gait MJ, Dickson G (2015). Combination antisense treatment for destructive exon skipping of myostatin and open reading frame rescue of dystrophin in neonatal mdx mice. Mol Ther.

[120] Béchir N, Pecchi E, Vilmen C, Le Fur Y, Amthor H, Bernard M (2016). ActRIIB blockade increases force-generating capacity and preserves energy supply in exercising mdx mouse muscle in vivo. FASEB J.

[121] Martin PT, Xu R, Rodino-Klapac LR, Oglesbay E, Camboni M, Montgomery CL (2009). Overexpression of Galgt2 in skeletal muscle prevents injury resulting from eccentric contractions in both mdx and wild-type mice. Am J Physiol Cell Physiol.

[122] Xu R, Chandrasekharan K, Yoon JH, Camboni M, Martin PT (2007). Overexpression of the cytotoxic T cell (CT) carbohydrate inhibits muscular dystrophy in the dyW mouse model of congenital muscular dystrophy 1A. Am J Pathol.

[123] Xu R, DeVries S, Camboni M, Martin PT (2009). Overexpression of Galgt2 reduces dystrophic pathology in the skeletal muscles of alpha sarcoglycan-deficient mice. Am J Pathol.

[124] Xu R, Jia Y, Zygmunt DA, Martin PT (2019). rAAVrh74.MCK.GALGT2 protects against loss of hemodynamic function in the aging mdx mouse heart. Mol Ther.

[125] Mercado ML, Amenta AR, Hagiwara H, Rafii MS, Lechner BE, Owens RT (2006). Biglycan regulates the expression and sarcolemmal localization of dystrobrevin, syntrophin, and nNOS. FASEB J.

[126] Amenta AR, Yilmaz A, Bogdanovich S, McKechnie BA, Abedi M, Khurana TS (2011). Biglycan recruits utrophin to the sarcolemma and counters dystrophic pathology in mdx mice. Proc Natl Acad Sci USA.

[127] Lai Y, Zhao J, Yue Y, Wasala NB, Duan D (2014). Partial restoration of cardiac function with ΔPDZ nNOS in aged mdx model of Duchenne cardiomyopathy. Hum Mol Genet.

[128] Goonasekera SA, Lam CK, Millay DP, Sargent MA, Hajjar RJ, Kranias EG (2011). Mitigation of muscular dystrophy in mice by SERCA overexpression in skeletal muscle. J Clin Investig.

[129] Morine KJ, Sleeper MM, Barton ER, Sweeney HL (2010). Overexpression of SERCA1a in the mdx diaphragm reduces susceptibility to contraction-induced damage. Hum Gene Ther.

[130] Shin J-H, Bostick B, Yue Y, Hajjar R, Duan D (2011). SERCA2a gene transfer improves electrocardiographic performance in aged mdx mice. J Transl Med.

[131] Wasala NB, Yue Y, Lostal W, Wasala LP, Niranjan N, Hajjar RJ (2020). Single SERCA2a therapy ameliorated dilated cardiomyopathy for 18 months in a mouse model of Duchenne muscular dystrophy. Mol Ther.

[132] Meng J, Chun S, Asfahani R, Lochmüller H, Muntoni F, Morgan J (2014). Human skeletal muscle-derived CD133+ cells form functional satellite cells after intramuscular transplantation in immunodeficient host mice. Mol Ther.

[133] Negroni E, Riederer I, Chaouch S, Belicchi M, Razini P, Di Santo J (2009). In vivo myogenic potential of human CD133+ muscle-derived stem cells: a quantitative study. Mol Ther.

[134] Torrente Y, Belicchi M, Sampaolesi M, Pisati F, Meregalli M, D’Antona G (2004). Human circulating AC133(+) stem cells restore dystrophin expression and ameliorate function in dystrophic skeletal muscle. J Clin Investig.

[135] Wakitani S, Saito T, Caplan AI (1995). Myogenic cells derived from rat bone marrow mesenchymal stem cells exposed to 5-azacytidine. Muscle Nerve.

[136] Ferrari G, Cusella G, Angelis D, Coletta M, Paolucci E, Stornaiuolo A (1998). Muscle regeneration by bone marrow-derived myogenic progenitors. Science.

[137] Grounds MD, Davies KE (2007). The allure of stem cell therapy for muscular dystrophy. Neuromuscul Disord.

[138] Bretag AH (2007). Stem cell treatment of dystrophic dogs. Nature.

[139] Mauro A (1961). Satellite cell of skeletal muscle fibers. J Biophys Biochem Cytol.

[140] Seale P, Sabourin LA, Girgis-Gabardo A, Mansouri A, Gruss P, Rudnicki MA (2000). Pax7 is required for the specification of myogenic satellite cells. Cell.

[141] Partridge TA, Morgan JE, Coulton GR, Hoffman EP, Kunkel LM (1989). Conversion of mdx myofibres from dystrophin-negative to -positive by injection of normal myoblasts. Nature.

[142] Karpati G, Pouliot Y, Zubrzycka-Gaarn E, Carpenter S, Ray PN, Worton RG (1989). Dystrophin is expressed in mdx skeletal muscle fibers after normal myoblast implantation. Am J Pathol.

[143] Hagiwara Y, Mizuno Y, Takemitsu M, Matsuzaki T, Nonaka I, Ozawa E (1995). Dystrophin-positive muscle fibers following C2 myoblast transplantation into mdx nude mice. Acta Neuropathol.

[144] Mendell JR, Kissel JT, Amato AA, King W, Signore L, Prior TW (1995). Myoblast transfer in the treatment of Duchenne’s muscular dystrophy. N Engl J Med.

[145] Miller RG, Sharma KR, Pavlath GK, Gussoni E, Mynhier M, Lanctot AM (1997). Myoblast implantation in Duchenne muscular dystrophy: the San Francisco study. Muscle Nerve.

[146] Skuk D, Roy B, Goulet M, Chapdelaine P, Bouchard J-P, Roy R (2004). Dystrophin expression in myofibers of Duchenne muscular dystrophy patients following intramuscular injections of normal myogenic cells. Mol Ther.

[147] Skuk D, Goulet M, Roy B, Piette V, Côté CH, Chapdelaine P (2007). First test of a “high-density injection” protocol for myogenic cell transplantation throughout large volumes of muscles in a Duchenne muscular dystrophy patient: eighteen months follow-up. Neuromuscul Disord.

[148] Skuk D, Goulet M, Roy B, Chapdelaine P, Bouchard J-P, Roy R (2006). Dystrophin expression in muscles of duchenne muscular dystrophy patients after high-density injections of normal myogenic cells. J Neuropathol Exp Neurol.

[149] Dumont NA, Bentzinger CF, Sincennes M-C, Rudnicki MA (2015). Satellite cells and skeletal muscle regeneration. Compr Physiol.

[150] Minasi MG, Riminucci M, De Angelis L, Borello U, Berarducci B, Innocenzi A (2002). The meso-angioblast: a multipotent, self-renewing cell that originates from the dorsal aorta and differentiates into most mesodermal tissues. Development.

[151] Sampaolesi M, Torrente Y, Innocenzi A, Tonlorenzi R, D’Antona G, Pellegrino MA (2003). Cell therapy of α-sarcoglycan null dystrophic mice through intra-arterial delivery of mesoangioblasts. Science.

[152] Sampaolesi M, Blot S, D’Antona G, Granger N, Tonlorenzi R, Innocenzi A (2006). Mesoangioblast stem cells ameliorate muscle function in dystrophic dogs. Nature.

[153] Tajbakhsh S (2009). Skeletal muscle stem cells in developmental versus regenerative myogenesis. J Intern Med.

[154] Tedesco FS, Dellavalle A, Diaz-Manera J, Messina G, Cossu G (2010). Repairing skeletal muscle: regenerative potential of skeletal muscle stem cells. J Clin Investig.

[155] Tedesco FS, Hoshiya H, D’Antona G, Gerli MFM, Messina G, Antonini S (2011). Stem cell-mediated transfer of a human artificial chromosome ameliorates muscular dystrophy. Sci Transl Med.

[156] Iyer PS, Mavoungou LO, Ronzoni F, Zemla J, Schmid-Siegert E, Antonini S (2018). Autologous cell therapy approach for duchenne muscular dystrophy using PiggyBac transposons and mesoangioblasts. Mol Ther.

[157] Ley D, Van Zwieten R, Puttini S, Iyer P, Cochard A, Mermod N (2014). A PiggyBac-mediated approach for muscle gene transfer or cell therapy. Stem Cell Res.

[158] Cossu G, Previtali SC, Napolitano S, Cicalese MP, Tedesco FS, Nicastro F (2015). Intra-arterial transplantation of HLA-matched donor mesoangioblasts in Duchenne muscular dystrophy. EMBO Mol Med.

[159] Schneider JS, Vitale JM, Terzic A, Fraidenraich D (2009). Blastocyst injection of embryonic stem cells: a simple approach to unveil mechanisms of corrections in mouse models of human disease. Stem Cell Rev Rep.

[160] Darabi R, Arpke RW, Irion S, Dimos JT, Grskovic M, Kyba M (2012). Human ES- and iPS-derived myogenic progenitors restore DYSTROPHIN and improve contractility upon transplantation in dystrophic mice. Cell Stem Cell.

[161] Takahashi K, Tanabe K, Ohnuki M, Narita M, Ichisaka T, Tomoda K (2007). Induction of pluripotent stem cells from adult human fibroblasts by defined factors. Cell.

[162] Konieczny P, Swiderski K, Chamberlain JS (2013). Gene and cell-mediated therapies for muscular dystrophy. Muscle Nerve.

[163] Bellin M, Marchetto MC, Gage FH, Mummery CL (2012). Induced pluripotent stem cells: the new patient?. Nat Rev Mol Cell Biol.

[164] Swanson E, Wallace WD (2014). Handling and interpretation of heart transplant biopsies. Methods Mol Biol.

[165] Bellin M, Mummery CL (2016). Inherited heart disease—what can we expect from the second decade of human iPS cell research?. FEBS Lett.

[166] Filareto A, Parker S, Darabi R, Borges L, Iacovino M, Schaaf T (2013). An ex vivo gene therapy approach to treat muscular dystrophy using inducible pluripotent stem cells. Nat Commun.

[167] Kyrychenko V, Kyrychenko S, Tiburcy M, Shelton JM, Long C, Schneider JW (2017). Functional correction of dystrophin actin binding domain mutations by genome editing. JCI Insight.

[168] Pioner JM, Guan X, Klaiman JM, Racca AW, Pabon L, Muskheli V (2019). Absence of full-length dystrophin impairs normal maturation and contraction of cardiomyocytes derived from human induced pluripotent stem cells. Cardiovasc Res.

[169] Li HL, Fujimoto N, Sasakawa N, Shirai S, Ohkame T, Sakuma T (2014). Precise correction of the dystrophin gene in Duchenne muscular dystrophy patient induced pluripotent stem cells by TALEN and CRISPR-Cas9. Stem Cell Rep.

[170] Romagnuolo R, Masoudpour H, Porta-Sánchez A, Qiang B, Barry J, Laskary A (2019). Human embryonic stem cell-derived cardiomyocytes regenerate the infarcted pig heart but induce ventricular tachyarrhythmias. Stem Cell Rep.

[171] Liu Y-W, Chen B, Yang X, Fugate JA, Kalucki FA, Futakuchi-Tsuchida A (2018). Human embryonic stem cell-derived cardiomyocytes restore function in infarcted hearts of non-human primates. Nat Biotechnol.

[172] Makkar RR, Smith RR, Cheng K, Malliaras K, Thomson LE, Berman D (2012). Intracoronary cardiosphere-derived cells for heart regeneration after myocardial infarction (CADUCEUS): a prospective, randomised phase 1 trial. Lancet.

[173] Chakravarty T, Makkar R, Henry T, Kittleson M, Friedman J, Berman D (2019). TCT-820 multivessel intracoronary infusion of allogeneic cardiosphere derived cells in dilated cardiomyopathy: long term outcomes of the dilated cardiomyopathy intervention with allogeneic myocardially-regenerative cells (DYNAMIC Trial). J Am Coll Cardiol.

[174] Aminzadeh MA, Rogers RG, Fournier M, Tobin RE, Guan X, Childers MK (2018). Exosome-mediated benefits of cell therapy in mouse and human models of Duchenne muscular dystrophy. Stem Cell Rep.

[175] Taylor M, Jefferies J, Byrne B, Lima J, Ambale-Venkatesh B, Ostovaneh MR (2019). Cardiac and skeletal muscle effects in the randomized HOPE-Duchenne trial. Neurology.

[176] Maliken BD, Molkentin JD (2018). Undeniable evidence that the adult mammalian heart lacks an endogenous regenerative stem cell. Circulation.

[177] Kretzschmar K, Post Y, Bannier-Hélaouët M, Mattiotti A, Drost J, Basak O (2018). Profiling proliferative cells and their progeny in damaged murine hearts. Proc Natl Acad Sci USA.

[178] Vagnozzi RJ, Sargent MA, Lin S-CJ, Palpant NJ, Murry CE, Molkentin JD (2018). Genetic lineage tracing of Sca-1+ cells reveals endothelial but not myogenic contribution to the murine heart. Circulation.

[179] Huard C, Moisset PA, Dicaire A, Merly F, Tardif F, Asselin I (1998). Transplantation of dermal fibroblasts expressing MyoD1 in mouse muscles. Biochem Biophys Res Commun.

[180] Lattanzi L, Salvatori G, Coletta M, Sonnino C, De Angelis MGC, Gioglio L (1998). High efficiency myogenic conversion of human fibroblasts by adenoviral vector-mediated MyoD gene transfer. An alternative strategy for ex vivo gene therapy of primary myopathies. J Clin Investig.

[181] Kimura E, Han JJ, Li S, Fall B, Ra J, Haraguchi M (2008). Cell-lineage regulated myogenesis for dystrophin replacement: a novel therapeutic approach for treatment of muscular dystrophy. Hum Mol Genet.

[182] Ito N, Kii I, Shimizu N, Tanaka H, Takeda S (2017). Direct reprogramming of fibroblasts into skeletal muscle progenitor cells by transcription factors enriched in undifferentiated subpopulation of satellite cells. Sci Rep.

[183] Lee E-J, Kim M, Kim YD, Chung M-J, Elfadl A, Ulah HMA (2018). Establishment of stably expandable induced myogenic stem cells by four transcription factors. Cell Death Dis.

[184] Bar-Nur O, Gerli MFM, Di Stefano B, Almada AE, Galvin A, Coffey A (2018). Direct reprogramming of mouse fibroblasts into functional skeletal muscle progenitors. Stem Cell Rep.

[185] Guiraud S, Davies KE (2017). Pharmacological advances for treatment in Duchenne muscular dystrophy. Curr Opin Pharmacol.

[186] Spinazzola JM, Kunkel LM (2016). Pharmacological therapeutics targeting the secondary defects and downstream pathology of Duchenne muscular dystrophy. Expert Opin Orphan Drugs.

[187] Schäcke H, Döcke WD, Asadullah K (2002). Mechanisms involved in the side effects of glucocorticoids. Pharmacol Ther.

[188] Biggar WD, Harris VA, Eliasoph L, Alman B (2006). Long-term benefits of deflazacort treatment for boys with Duchenne muscular dystrophy in their second decade. Neuromuscul Disord.

[189] Miyatake S, Shimizu-Motohashi Y, Takeda S, Aoki Y (2016). Anti-inflammatory drugs for Duchenne muscular dystrophy: focus on skeletal muscle-releasing factors. Drug Des Dev Ther.

[190] Quattrocelli M, Barefield DY, Warner JL, Vo AH, Hadhazy M, Earley JU (2017). Intermittent glucocorticoid steroid dosing enhances muscle repair without eliciting muscle atrophy. J Clin Investig.

[191] Mavrogeni S, Papavasiliou A, Douskou M, Kolovou G, Papadopoulou E, Cokkinos DV (2009). Effect of deflazacort on cardiac and sternocleidomastoid muscles in Duchenne muscular dystrophy: a magnetic resonance imaging study. Eur J Paediatr Neurol.

[192] Schram G, Fournier A, Leduc H, Dahdah N, Therien J, Vanasse M (2013). All-cause mortality and cardiovascular outcomes with prophylactic steroid therapy in Duchenne muscular dystrophy. J Am Coll Cardiol.

[193] Silversides CK, Webb GD, Harris VA, Biggar DW (2003). Effects of deflazacort on left ventricular function in patients with Duchenne muscular dystrophy. Am J Cardiol.

[194] Heier CR, Damsker JM, Yu Q, Dillingham BC, Huynh T, Van der Meulen JH (2013). VBP15, a novel anti-inflammatory and membrane-stabilizer, improves muscular dystrophy without side effects. EMBO Mol Med.

[195] Hoffman EP, Riddle V, Siegler MA, Dickerson D, Backonja M, Kramer WG (2018). Phase 1 trial of vamorolone, a first-in-class steroid, shows improvements in side effects via biomarkers bridged to clinical outcomes. Steroids.

[196] Hoffman EP, Schwartz BD, Mengle-Gaw LJ, Smith EC, Castro D, Mah JK (2019). Vamorolone trial in Duchenne muscular dystrophy shows dose-related improvement of muscle function. Neurology.

[197] Conklin LS, Damsker JM, Hoffman EP, Jusko WJ, Mavroudis PD, Schwartz BD (2018). Phase IIa trial in Duchenne muscular dystrophy shows vamorolone is a first-in-class dissociative steroidal anti-inflammatory drug. Pharmacol Res.

[198] Heier CR, Yu Q, Fiorillo AA, Tully CB, Tucker A, Mazala DA (2019). Vamorolone targets dual nuclear receptors to treat inflammation and dystrophic cardiomyopathy. Life Sci Alliance.

[199] Minetti GC, Colussi C, Adami R, Serra C, Mozzetta C, Parente V (2006). Functional and morphological recovery of dystrophic muscles in mice treated with deacetylase inhibitors. Nat Med.

[200] Colussi C, Mozzetta C, Gurtner A, Illi B, Rosati J, Straino S (2008). HDAC2 blockade by nitric oxide and histone deacetylase inhibitors reveals a common target in Duchenne muscular dystrophy treatment. PNAS.

[201] Consalvi S, Mozzetta C, Bettica P, Germani M, Fiorentini F, Del Bene F (2013). Preclinical studies in the mdx mouse model of duchenne muscular dystrophy with the histone deacetylase inhibitor givinostat. Mol Med.

[202] Iezzi S, Di Padova M, Serra C, Caretti G, Simone C, Maklan E (2004). Deacetylase inhibitors increase muscle cell size by promoting myoblast recruitment and fusion through induction of follistatin. Dev Cell.

[203] Mendell JR, Sahenk Z, Malik V, Gomez AM, Flanigan KM, Lowes LP (2015). A phase 1/2a follistatin gene therapy trial for becker muscular dystrophy. Mol Ther.

[204] Colussi C, Banfi C, Brioschi M, Tremoli E, Straino S, Spallotta F (2010). Proteomic profile of differentially expressed plasma proteins from dystrophic mice and following suberoylanilide hydroxamic acid treatment. Proteom Clin Appl.

[205] Bettica P, Petrini S, D’Oria V, D’Amico A, Catteruccia M, Pane M (2016). Histological effects of givinostat in boys with Duchenne muscular dystrophy. Neuromuscul Disord.

[206] Muntoni F, Tejura B, Spinty S, Roper H, Hughes I, Layton G (2019). A phase 1b trial to assess the pharmacokinetics of ezutromid in pediatric duchenne muscular dystrophy patients on a balanced diet. Clin Pharmacol Drug Dev.

[207] Guiraud S, Squire SE, Edwards B, Chen H, Burns DT, Shah N (2015). Second-generation compound for the modulation of utrophin in the therapy of DMD. Hum Mol Genet.

[208] Moorwood C, Lozynska O, Suri N, Napper AD, Diamond SL, Khurana TS (2011). Drug discovery for Duchenne muscular dystrophy via utrophin promoter activation screening. PLoS One.

[209] Duboc D, Meune C, Pierre B, Wahbi K, Eymard B, Toutain A (2007). Perindopril preventive treatment on mortality in Duchenne muscular dystrophy: 10 years’ follow-up. Am Heart J.

[210] Dikalov SI, Nazarewicz RR (2013). Angiotensin II-induced production of mitochondrial reactive oxygen species: potential mechanisms and relevance for cardiovascular disease. Antioxid Redox Signal.

[211] Bangalore S, Fakheri R, Toklu B, Ogedegbe G, Weintraub H, Messerli FH (2016). Angiotensin-converting enzyme inhibitors or angiotensin receptor blockers in patients without heart failure? Insights from 254,301 patients from randomized trials. Mayo Clin Proc.

[212] Kajimoto H, Ishigaki K, Okumura K, Tomimatsu H, Nakazawa M, Saito K (2006). Beta-blocker therapy for cardiac dysfunction in patients with muscular dystrophy. Circ J.

[213] Viollet L, Thrush PT, Flanigan KM, Mendell JR, Allen HD (2012). Effects of angiotensin-converting enzyme inhibitors and/or beta blockers on the cardiomyopathy in Duchenne muscular dystrophy. Am J Cardiol.

[214] Bourke JP, Watson G, Muntoni F, Spinty S, Roper H, Guglieri M (2018). Randomised placebo-controlled trial of combination ACE inhibitor and beta-blocker therapy to prevent cardiomyopathy in children with Duchenne muscular dystrophy? (DMD Heart Protection Study): a protocol study. BMJ Open.

[215] Raman SV, Hor KN, Mazur W, Halnon NJ, Kissel JT, He X (2015). Eplerenone for early cardiomyopathy in Duchenne muscular dystrophy: a randomised, double-blind, placebo-controlled trial. Lancet Neurol.

[216] Sander M, Chavoshan B, Harris SA, Iannaccone ST, Stull JT, Thomas GD (2000). Functional muscle ischemia in neuronal nitric oxide synthase-deficient skeletal muscle of children with Duchenne muscular dystrophy. Proc Natl Acad Sci USA.

[217] Szabo C (2017). Hydrogen sulfide, an enhancer of vascular nitric oxide signaling: mechanisms and implications. Am J Physiol Cell Physiol.

[218] Percival JM, Whitehead NP, Adams ME, Adamo CM, Beavo JA, Froehner SC (2012). Sildenafil reduces respiratory muscle weakness and fibrosis in the mdx mouse model of Duchenne muscular dystrophy. J Pathol.

[219] Hammers DW, Sleeper MM, Forbes SC, Shima A, Walter GA, Sweeney HL (2016). Tadalafil treatment delays the onset of cardiomyopathy in dystrophin-deficient hearts. J Am Heart Assoc.

[220] Nelson MD, Rader F, Tang X, Tavyev J, Nelson SF, Miceli MC (2014). PDE5 inhibition alleviates functional muscle ischemia in boys with Duchenne muscular dystrophy. Neurology.

[221] Victor RG, Sweeney HL, Finkel R, McDonald CM, Byrne B, Eagle M (2017). A phase 3 randomized placebo-controlled trial of tadalafil for Duchenne muscular dystrophy. Neurology.

[222] Harricane MC, Fabbrizio E, Lees D, Prades C, Travo P, Mornet D (1994). Dystrophin does not influence regular cytoskeletal architecture but is required for contractile performance in smooth muscle aortic cells. Cell Biol Int.

[223] Straino S, Germani A, Di Carlo A, Porcelli D, De Mori R, Mangoni A (2004). Enhanced arteriogenesis and wound repair in dystrophin-deficient mdx mice. Circulation.

[224] Bronisz-Budzyńska I, Chwalenia K, Mucha O, Podkalicka P, Józkowicz A (2019). miR-146a deficiency does not aggravate muscular dystrophy in mdx mice. Skelet Muscle.

[225] Messina S, Mazzeo A, Bitto A, Aguennouz M, Migliorato A, De Pasquale MG (2007). VEGF overexpression via adeno-associated virus gene transfer promotes skeletal muscle regeneration and enhances muscle function in mdx mice. FASEB J.

[226] Loboda A, Damulewicz M, Pyza E, Jozkowicz A, Dulak J (2016). Role of Nrf2/HO-1 system in development, oxidative stress response and diseases: an evolutionarily conserved mechanism. Cell Mol Life Sci.

[227] Loboda A, Jozkowicz A, Dulak J (2015). HO-1/CO system in tumor growth, angiogenesis and metabolism—targeting HO-1 as an anti-tumor therapy. Vasc Pharmacol.

[228] Loboda A, Jazwa A, Grochot-Przeczek A, Rutkowski AJ, Cisowski J, Agarwal A (2008). Heme oxygenase-1 and the vascular bed: from molecular mechanisms to therapeutic opportunities. Antioxid Redox Signal.

[229] Dulak J, Józkowicz A, Foresti R, Kasza A, Frick M, Huk I (2002). Heme oxygenase activity modulates vascular endothelial growth factor synthesis in vascular smooth muscle cells. Antioxid Redox Signal.

[230] Józkowicz A, Huk I, Nigisch A, Weigel G, Dietrich W, Motterlini R (2003). Heme oxygenase and angiogenic activity of endothelial cells: stimulation by carbon monoxide and inhibition by tin protoporphyrin-IX. Antioxid Redox Signal.

[231] Deshane J, Chen S, Caballero S, Grochot-Przeczek A, Was H, Li Calzi S (2007). Stromal cell-derived factor 1 promotes angiogenesis via a heme oxygenase 1-dependent mechanism. J Exp Med.

[232] Skrzypek K, Tertil M, Golda S, Ciesla M, Weglarczyk K, Collet G (2013). Interplay between heme oxygenase-1 and miR-378 affects non-small cell lung carcinoma growth, vascularization, and metastasis. Antioxid Redox Signal.

[233] Tertil M, Golda S, Skrzypek K, Florczyk U, Weglarczyk K, Kotlinowski J (2015). Nrf2-heme oxygenase-1 axis in mucoepidermoid carcinoma of the lung: antitumoral effects associated with down-regulation of matrix metalloproteinases. Free Radic Biol Med.

[234] Loboda A, Was H, Jozkowicz A, Dulak J (2008). Janus face of Nrf2-HO-1 axis in cancer-friend in chemoprevention, foe in anticancer therapy. Lung Cancer.

[235] Was H, Cichon T, Smolarczyk R, Rudnicka D, Stopa M, Chevalier C (2006). Overexpression of heme oxygenase-1 in murine melanoma: increased proliferation and viability of tumor cells, decreased survival of mice. Am J Pathol.

[236] Grochot-Przeczek A, Kotlinowski J, Kozakowska M, Starowicz K, Jagodzinska J, Stachurska A (2014). Heme oxygenase-1 is required for angiogenic function of bone marrow-derived progenitor cells: role in therapeutic revascularization. Antioxid Redox Signal.

[237] Jazwa A, Stepniewski J, Zamykal M, Jagodzinska J, Meloni M, Emanueli C (2013). Pre-emptive hypoxia-regulated HO-1 gene therapy improves post-ischaemic limb perfusion and tissue regeneration in mice. Cardiovasc Res.

[238] Kozakowska M, Ciesla M, Stefanska A, Skrzypek K, Was H, Jazwa A (2012). Heme oxygenase-1 inhibits myoblast differentiation by targeting myomirs. Antioxid Redox Signal.

[239] Kozakowska M, Pietraszek-Gremplewicz K, Ciesla M, Seczynska M, Bronisz-Budzynska I, Podkalicka P (2018). Lack of heme oxygenase-1 induces inflammatory reaction and proliferation of muscle satellite cells after cardiotoxin-induced skeletal muscle injury. Am J Pathol.

[240] Pietraszek-Gremplewicz K, Kozakowska M, Bronisz-Budzynska I, Ciesla M, Mucha O, Podkalicka P (2018). Heme oxygenase-1 influences satellite cells and progression of Duchenne muscular dystrophy in mice. Antioxid Redox Signal.

[241] Terrill JR, Radley-Crabb HG, Grounds MD, Arthur PG (2012). N-Acetylcysteine treatment of dystrophic mdx mice results in protein thiol modifications and inhibition of exercise induced myofibre necrosis. Neuromuscul Disord.

[242] Whitehead NP, Pham C, Gervasio OL, Allen DG (2008). N-Acetylcysteine ameliorates skeletal muscle pathophysiology in mdx mice. J Physiol (Lond).

[243] Pinniger GJ, Terrill JR, Assan EB, Grounds MD, Arthur PG (2017). Pre-clinical evaluation of *N*-acetylcysteine reveals side effects in the mdx mouse model of Duchenne muscular dystrophy. J Physiol (Lond).

[244] Buetler TM, Renard M, Offord EA, Schneider H, Ruegg UT (2002). Green tea extract decreases muscle necrosis in mdx mice and protects against reactive oxygen species. Am J Clin Nutr.

[245] Dorchies OM, Wagner S, Vuadens O, Waldhauser K, Buetler TM, Kucera P (2006). Green tea extract and its major polyphenol (-)-epigallocatechin gallate improve muscle function in a mouse model for Duchenne muscular dystrophy. Am J Physiol Cell Physiol.

[246] Fenichel GM, Brooke MH, Griggs RC, Mendell JR, Miller JP, Moxley RT (1988). Clinical investigation in Duchenne muscular dystrophy: penicillamine and vitamin E. Muscle Nerve.

[247] Walton JN, Nattrass FJ (1954). On the classification, natural history and treatment of the myopathies. Brain.

[248] Berneske GM, Butson AR, Gauld EN, Levy D (1960). Clinical trial of high dosage vitamin E in human muscular dystrophy. Can Med Assoc J.

[249] Kim J-H, Kwak H-B, Thompson LV, Lawler JM (2013). Contribution of oxidative stress to pathology in diaphragm and limb muscles with Duchenne muscular dystrophy. J Muscle Res Cell Motil.

[250] Whitehead NP, Kim MJ, Bible KL, Adams ME, Froehner SC (2015). A new therapeutic effect of simvastatin revealed by functional improvement in muscular dystrophy. Proc Natl Acad Sci USA.

[251] Gazzerro P, Proto MC, Gangemi G, Malfitano AM, Ciaglia E, Pisanti S (2012). Pharmacological actions of statins: a critical appraisal in the management of cancer. Pharmacol Rev.

[252] Ezad S, Cheema H, Collins N (2018). Statin-induced rhabdomyolysis: a complication of a commonly overlooked drug interaction. Oxf Med Case Rep.

[253] Mendes P, Robles PG, Mathur S (2014). Statin-induced rhabdomyolysis: a comprehensive review of case reports. Physiother Can.

[254] Whitehead NP (2016). Enhanced autophagy as a potential mechanism for the improved physiological function by simvastatin in muscular dystrophy. Autophagy.

[255] Frick M, Dulak J, Cisowski J, Józkowicz A, Zwick R, Alber H (2003). Statins differentially regulate vascular endothelial growth factor synthesis in endothelial and vascular smooth muscle cells. Atherosclerosis.

[256] Weis M (2002). Statins have biphasic effects on angiogenesis. Circulation.

[257] Dulak J, Loboda A, Jazwa A, Zagorska A, Dörler J, Alber H (2005). Atorvastatin affects several angiogenic mediators in human endothelial cells. Endothelium.

[258] Davignon J (2004). The cardioprotective effects of statins. Curr Atheroscler Rep.

[259] Kim MJ, Bible KL, Regnier M, Adams ME, Froehner SC, Whitehead NP (2019). Simvastatin provides long-term improvement of left ventricular function and prevents cardiac fibrosis in muscular dystrophy. Physiol Rep.

[260] Iwere RB, Hewitt J (2015). Myopathy in older people receiving statin therapy: a systematic review and meta-analysis. Br J Clin Pharmacol.

[261] Parker BA, Thompson PD (2012). Effect of statins on skeletal muscle: exercise, myopathy, and muscle outcomes. Exerc Sport Sci Rev.

[262] Bhardwaj S, Selvarajah S, Schneider EB (2013). Muscular effects of statins in the elderly female: a review. Clin Interv Aging.

[263] Friedman RC, Farh KK-H, Burge CB, Bartel DP (2009). Most mammalian mRNAs are conserved targets of microRNAs. Genome Res.

[264] D’Agostino M, Torcinaro A, Madaro L, Marchetti L, Sileno S, Beji S (2018). Role of miR-200c in myogenic differentiation impairment via p66Shc: implication in skeletal muscle regeneration of dystrophic mdx mice. Oxid Med Cell Longev.

[265] Podkalicka P, Mucha O, Bronisz-Budzyńska I, Kozakowska M, Pietraszek-Gremplewicz K, Cetnarowska A (2020). Lack of miR-378 attenuates muscular dystrophy in mdx mice. JCI Insight.

[266] Wei X, Li H, Zhang B, Li C, Dong D, Lan X (2016). miR-378a-3p promotes differentiation and inhibits proliferation of myoblasts by targeting HDAC4 in skeletal muscle development. RNA Biol.

[267] Proctor CJ, Goljanek-Whysall K (2017). Using computer simulation models to investigate the most promising microRNAs to improve muscle regeneration during ageing. Sci Rep.

[268] Gagan J, Dey BK, Layer R, Yan Z, Dutta A (2011). MicroRNA-378 targets the myogenic repressor MyoR during myoblast differentiation. J Biol Chem.

[269] Krist B, Podkalicka P, Mucha O, Mendel M, Sępioł A, Rusiecka OM (2019). miR-378a influences vascularization in skeletal muscles. Cardiovasc Res.

[270] Verma M, Asakura Y, Asakura A (2019). Inhibition of microRNA-92a increases blood vessels and satellite cells in skeletal muscle but does not improve duchenne muscular dystrophy-related phenotype in mdx mice. Muscle Nerve.

[271] Zschüntzsch J, Zhang Y, Klinker F, Makosch G, Klinge L, Malzahn D (2016). Treatment with human immunoglobulin G improves the early disease course in a mouse model of Duchenne muscular dystrophy. J Neurochem.

[272] Zschüntzsch J, Jouvenal PV, Zhang Y, Klinker F, Tiburcy M, Liebetanz D (2020). Long-term human IgG treatment improves heart and muscle function in a mouse model of Duchenne muscular dystrophy. J Cachexia Sarcopenia Muscle.

[273] McMurray JJV, Adamopoulos S, Anker SD, Auricchio A, Böhm M, Dickstein K (2012). ESC guidelines for the diagnosis and treatment of acute and chronic heart failure 2012: The Task Force for the Diagnosis and Treatment of Acute and Chronic Heart Failure 2012 of the European Society of Cardiology. Developed in collaboration with the Heart Failure Association (HFA) of the ESC. Eur J Heart Fail.

[274] Boccanegra B, Verhaart IEC, Cappellari O, Vroom E, De Luca A (2020). Safety issues and harmful pharmacological interactions of nutritional supplements in Duchenne muscular dystrophy: considerations for Standard of Care and emerging virus outbreaks. Pharmacol Res.

[275] Verhaart IEC, van den Engel-Hoek L, Fiorotto ML, Franken-Verbeek M, Vroom E (2018). Workshop participants. Nutrition in Duchenne muscular dystrophy 16–18, Zaandam, the Netherlands. Neuromuscul Disord.

[276] Sipp D, Caulfield T, Kaye J, Barfoot J, Blackburn C, Chan S (2017). Marketing of unproven stem cell-based interventions: a call to action. Sci Transl Med.

[277] Sipp D, Robey PG, Turner L (2018). Clear up this stem-cell mess. Nature.

[278] Langrzyk A, Nowak WN, Stępniewski J, Jaźwa A, Florczyk-Soluch U, Józkowicz A (2018). Critical view on mesenchymal stromal cells in regenerative medicine. Antioxid Redox Signal.

